# Unlocking the *in vivo* therapeutic potential of radiation-activated photodynamic therapy for locally advanced rectal cancer with lymph node involvement

**DOI:** 10.1016/j.ebiom.2025.105724

**Published:** 2025-05-12

**Authors:** Rui Sang, Sheri Nixdorf, Tzongtyng Hung, Carl Power, Fei Deng, Thuy Anh Bui, Alexander Engel, Ewa M. Goldys, Wei Deng

**Affiliations:** a Graduate School of Biomedical Engineering, ARC Centre of Excellence in Nanoscale Biophotonics, Faculty of Engineering, University of New South Wales, Sydney, NSW, 2052, Australia; b School of Biomedical Engineering, University of Technology Sydney, Sydney, NSW, 2007, Australia; c Biological Resources Imaging Laboratory, Mark Wainwright Analytical Centre, University of New South Wales, Sydney, NSW, 2052, Australia; d Sydney Medical School, University of Sydney, Sydney, NSW, 2050, Australia; e Department of Colorectal Surgery, Royal North Shore Hospital, St Leonards, Sydney, NSW, 2065, Australia

**Keywords:** Locally advanced rectal cancer, Lymph node involvement, Low dose radiation, Photodynamic therapy, Reactive oxygen species

## Abstract

**Background:**

Rectal cancer is a leading cause of cancer-related mortality worldwide. The recurrence of locally advanced rectal cancer (LARC), particularly in cases involving lymph node-positive tumours, remains a critical challenge in rectal cancer management. In this study, a therapeutic strategy, radiation-activated photodynamic therapy (RA-PDT), for the treatment of LARC with lymph node-positive tumours was developed and evaluated.

**Methods:**

RA-PDT was achieved by using a lipid-polymer hybrid nanoplatform loaded with verteporfin (VP) and functionalised with folic acid (FA) as a targeting molecule. Upon receiving a single 4 Gy fraction of radiation, VP was effectively activated, generating sufficient reactive oxygen species (ROS) to induce cancer cell death–however surrounding tissue was less affected and was spared. The efficacy of this strategy was assessed through *in vitro* cytotoxicity studies in HCT116 cells, as well as in orthotopic and subcutaneous mouse models. *In vivo* lymph node tumour progression was also evaluated.

**Findings:**

RA-PDT effectively generated ROS following 4 Gy irradiation and exhibited significant cytotoxicity in HCT116 cells. *In vivo*, this strategy largely inhibited primary tumour growth in both orthotopic and subcutaneous mouse models while also suppressing lymph node tumour progression. Surrounding tissues were minimally affected, highlighting the precision and safety of this approach.

**Interpretation:**

RA-PDT demonstrates potential as a safe therapeutic strategy for LARC, paving the way for its clinical translation.

**Funding:**

This study was supported by the 10.13039/501100000925Australian National Health and Medical Research Council (GNT1181889), fellowship award (2019/CDF1013) from 10.13039/501100001171Cancer Institute NSW, Australia, the 10.13039/100015360Australian Research Council Centre of Excellence for Nanoscale Biophotonics (CE140100003), UNSW SHARP funding, project grant from 10.13039/100008436National Foundation for Medical Research and Innovation, Australia, International Research Training Program Scholarship (IRTP) from Australian Government, PhD Research Scholar Award from Sydney Vital Translational Cancer Research, and Translational Cancer Research Network PhD Scholarship Top-up award.

**Research in context**

**Evidence before this study**

Our previous research has successfully demonstrated that VP can produce sufficient ROS upon exposure to low dose radiation both in solution and at the cellular level. This provided a solid foundation for the current study, which leverages VP's ROS-generating properties under irradiation to develop a treatment strategy for LARC with lymph node-positive tumours.

**Added value of this study**

This study presents a treatment strategy utilising a rationally designed nanocarrier system capable of efficiently delivering VP to cancer cells. Upon exposure to a low dose of radiation, VP is activated to produce sufficient ROS, which induced cancer cell death. Our findings demonstrated that this treatment method significantly inhibited primary tumour growth in both orthotopic and subcutaneous mouse models, along with the suppression of lymph node tumour progression *in vivo*.

**Implications of all the available evidence**

RA-PDT is free of systemic effects of conventional chemotherapy drugs, offering a paradigm-shifting alternative for patients who are unable to tolerate or have developed resistance to chemotherapy. Moreover, all components of this formulation are already clinically approved and are synthesised using a straightforward method. This rational design minimises the need for complex manufacturing steps, simplifying future scale-up processes. In addition, this approach may circumvent the translational challenges of RA-PDT by eliminating the reliance on metal nanomaterials, propelling RA-PDT closer to clinical translation, and implying a significant stride towards real-world applicability and patient benefit.

## Introduction

Colorectal cancer (CRC) remains the second leading cause of cancer-related deaths worldwide, with rectal cancer representing 30% of all colorectal cancers.^1^ The recurrence of locally advanced rectal cancer (LARC) remains a critical challenge in rectal cancer management, attributed to incomplete clearance of affected lymph nodes beyond the surgical site.^2^^,^^3^ Patients with LARC are typically treated or prevented from recurrence through neoadjuvant chemo/radiotherapy. However, radiotherapy and chemotherapy have dose-limiting toxicities, raising concerns about their limited efficacy and potential impact on bowel function and surrounding healthy tissue.^4^ Therefore, other methods are urgently needed that synergise with chemo/radiotherapy without raising toxicity, while also maximising bowel preservation.

In a standard photodynamic therapy (PDT) process, ROS are generated by activated photosensitisers under near infrared or visible light and they induce cancer cell death through apoptosis, autophagy, and necrosis mechanisms.^5^, ^6^, ^7^ However, this approach is not suitable for deep seated tumour treatment due to limited tissue penetration of light (less than 1 cm) used in PDT.^8^ To overcome this problem, radio-dynamic therapy has been developed, where instead of light, X-ray radiation is used to induce PDT effect. This approach is effective in deep tissue as radiation has well-known deep tissue penetrating properties.^9^^,^^10^ Previous investigations in this area employed scintillating nanoparticles to facilitate the conversion of X-ray photons into visible photons, thereby stimulating a nearby photosensitiser for ROS generation.^11^, ^12^, ^13^, ^14^, ^15^ For example, mesoporous LaF_3_:Tb has been demonstrated to convert X-ray energy into visible light which in turn activates co-loaded photosensitisers such as Rose Bengal for ROS generation.^11^^,^^12^ Silica/SrAl_2_O_4_:Eu nanoparticles have been reported to convert X-rays to green fluorescence, triggering co-loaded merocyanine 540 to produce singlet oxygen species (^1^O_2_), which induced 38% decrease in viability of radioresistant human glioblastoma cells (U87MG) and effective inhibition of subcutaneous U87MG tumour growth.^13^ In addition to scintillating nanoparticles, radiosensitisers were developed and used for RA-PDT to enhance ROS production and cancer cell killing effect.^16^, ^17^, ^18^, ^19^, ^20^, ^21^, ^22^, ^23^, ^24^ For example, nanoscale metal-organic frameworks (nMOFs) were acted as a radiosensitiser to generate hydroxyl radicals in the heavy metal secondary building units as well as to transfer energy absorbed from X-ray radiation to photosensitisers for enhancing ROS generation.^17^, ^18^, ^19^, ^20^ Gold nanoparticles strongly interact with X-ray radiation and directly generate some level of ROS.^21^, ^22^, ^23^ Polyoxomolybdate nanoclusters produce Auger electrons to directly induce DNA damage upon X-ray radiation.^24^ Copper-cysteamine (Cu-Cy) nanoparticles can be directly activated by X-ray to generate ROS for RA-PDT of CRC treatment.^25^ Nevertheless, these approaches rely on the utilisation of metal nanomaterials to facilitate ROS generation, which poses concerns regarding potential biological toxicity and other unfavourable effects, thereby imposing limitations on their translational applications.^26^

**Fig. 1:**
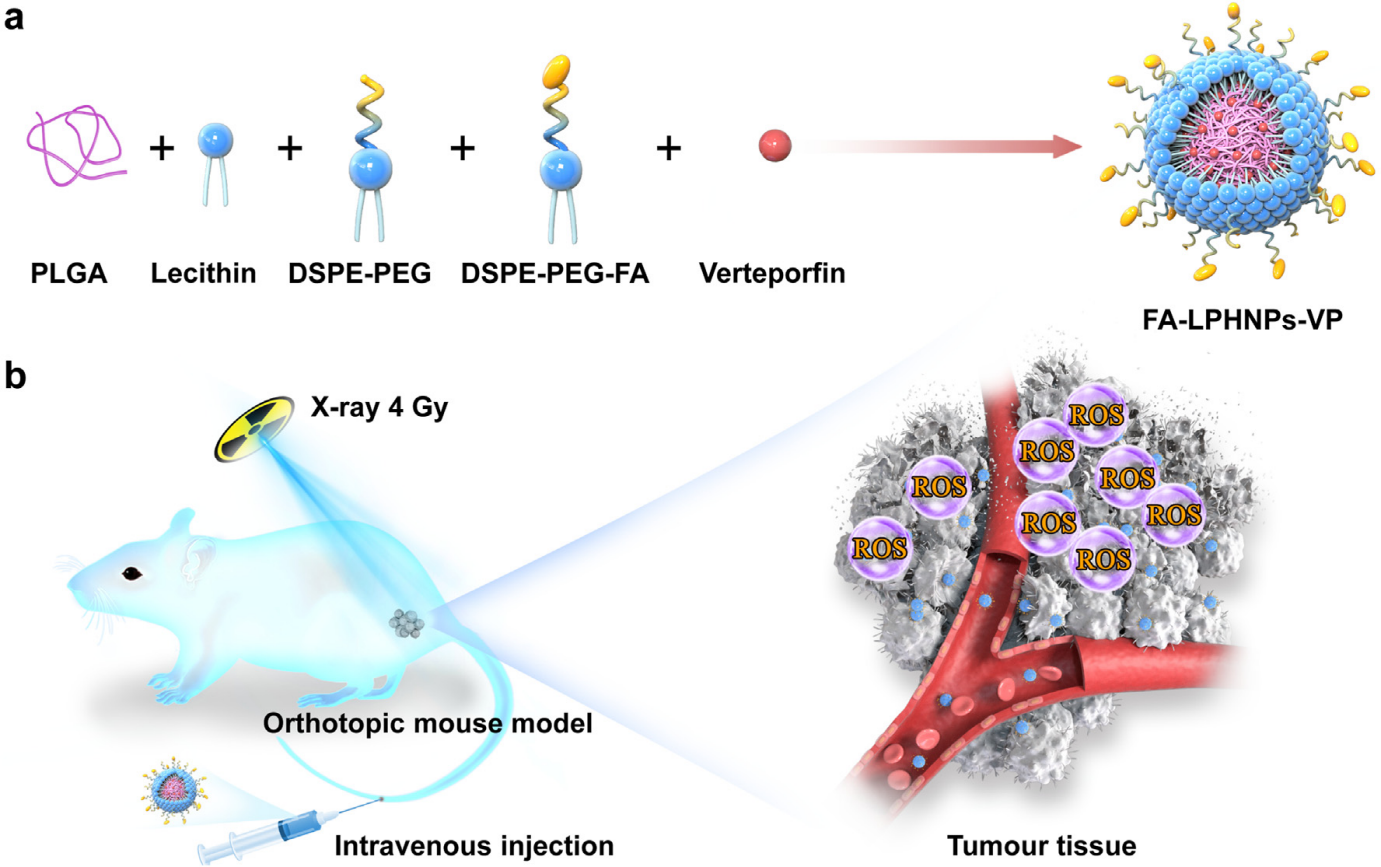
**The schematic illustration** of (a) the nanocarrier formulation and (b) RA-PDT effect on an orthotopic mouse model bearing rectal cancer.

Verteporfin (VP) is a clinically approved photosensitiser for age-related macular degeneration treatment.^27^ Our earlier findings demonstrated that VP can directly generate ROS at low doses of X-ray radiation.^21^^,^^22^^,^^28^^,^^29^ We hypothesised that this RA-PDT strategy where VP was directly activated by radiation can be used for controlling LARC with node-positive tumours in a mouse model. To prove this concept, we developed a lipid-polymer hybrid nanoplatform (LPHNP) carrying VP and targeting rectal cancer cells. The nanoparticle consists of lecithin and 1,2-distearoyl-snglycero-3-phosphoethanolamine-N-carboxy (polyethylene glycol) 2000 (DSPE-PEG2000), forming a lipid layer around the PLGA core. The inclusion of lecithin enhances biocompatibility and stability, while DSPE-PEG2000 extends the circulation time of the nanoparticles in the bloodstream.^30^^,^^31^ This formulation achieved the VP encapsulation efficiency up to over 90%. To enhance cancer cell targeting, the LPHNP surface was additionally modified with folic acid (FA) by incorporating DSPE-PEG-Folic acid (FA-LPHNPs-VP) into the composition.^32^ Fig. 1 illustrates the nanocarrier formulation and the *in vivo* RA-PDT effect on primary tumour.

In this study, we assessed the nanocarrier-enabled RA-PDT effect on the treatment of LARC. We first investigated the intracellular ROS generation, impact on the cellular apoptosis/necrosis pathways and *in vitro* RA-PDT effect in human colorectal cancer cells (HCT116). We further assessed the safety profile of drug formulation in an orthotopic mouse model bearing HCT116 cells. The evaluation included a histopathological examination of major organs, haematological analysis, and innate immune system assessment. The therapeutic impact of RA-PDT was determined through a comprehensive examination of growth of both primary and lymph node tumour, along with detailed histological and immunohistochemical analyses of the tumour tissues following treatment. Our findings indicated that this approach may circumvent the translational challenges of radio-dynamic therapy by eliminating the reliance on metal nanomaterials.

## Methods

### Study design

The aim of this study was to develop a RA-PDT strategy for treating LARC with node-tumour involvement, where the photosensitiser can be directly activated by radiation. First, folic acid-conjugated lipid-polymer hybrid nanoparticles loaded with verteporfin (FA-LPHNPs-VP) were synthesised and characterised. Intracellular ROS generation, the pathways of cellular apoptosis and necrosis, and the *in vitro* RA-PDT effects on CRC cells (HCT116) were then evaluated. The safety profile of the nanocarrier was assessed using an orthotopic mouse model. Finally, the RA-PDT effects were evaluated by monitoring tumour growth and lymph node metastasis progression in the orthotopic and lymph node metastasis mouse models model and conducting histological and immunohistochemical analysis of tumour tissues and lymph node tissues after treatment. Details on sampling replicates are provided in the figure legends.

### Nanocarrier synthesis and characterisation

The nanocarriers were prepared via self-assembly of lipids and PLGA via a modified nanoprecipitation method.^33^ In brief, lecithin (Avanti Polar Lipids, Cat#P7443)/DSPE-PEG2000 (Avanti Polar Lipids, Cat#880135P) (molar ratio: 8.5/1.5, for LPHNPs and LPHNPs-VP) or lecithin/DSPE-PEG2000/DSPE-PEG-FA (Nanocs Inc., Cat#PG2-DSFA-2k) (molar ratio: 8.5/1.125/0.375, for FA-LPHNPs-VP) were mixed in 4% ethanol aqueous solution then heated to 65 °C for dissolving lipids in a uniform fluid form. Next, acetonitrile containing PLGA (Sigma-Aldrich, Cat#719900) (0.71 mg/mL) and VP (Sigma-Aldrich, Cat#SML0534) (31.80 μM) were added dropwise into the preheated liquid solution while gently swirling, using a weight ratio of 1.8:1 of lipid/polymer. The solution was gentle stirred for 2 h at room temperature prior to evaporation of the solution to 1.5 mL by heating to 65 °C. The purification of nanoparticles was conducted using the Pur-A-Lyzer Maxi Dialysis Kit (Molecular weight cut-off 12–14 kDa; Sigma-Aldrich, Cat#PURX12015) to remove remaining free molecules. Nanoparticles were stored at 4 °C for further use. For characterisation, Zetasizer Nano-ZS (Malvern Panalytical Co, Malvern, UK) was used for nanoparticles size distribution and zeta potential measurement. The fluorescence spectra of VP were assessed via spectrofluorometer (FluoroMax-4 HORIBA Scientific Co, Kyoto, Japan). The absorption spectra were tested via spectrophotometer (Cary 5000 UV-Vis-NIR, Agilent Technologies, California, USA). The chemical compositions of FA-DSPE-PEG and nano-formulations were compared by using an Attenuated Total Reflectance-Fourier Transform Infrared Spectrometer (ATR-FTIR, Spectrum Two FTIR Spectrometer, PerkinElmer, Waltham, MA, USA) in the wavelength range of 4000–450 cm^−1^ with 4 cm^−1^ resolution. The morphology of prepared nanoparticles was observed under a transmission electron microscope (TEM) (JEOL 1400 Transmission Electron Microscope, JEOL Pty. Ltd, Tokyo, Japan). The stability of prepared nanoparticles in terms of size distribution and zeta potential was monitored for 4 weeks at room temperature. The VP encapsulation efficiency (EE%) in prepared nanoparticles was calculated as follows:

$$\text{EE}\%={\text{VP}}_{\text{loaded}}/{\text{VP}}_{\text{total}\;}\times \;100\%$$

The amount of VP loaded in nanoparticles (VP_loaded_) was obtained through the measurement of fluorescence signal of VP (Excitation/Emission: 425 nm/690 nm) after nanoparticles were completely dissolved in acetonitrile and calculation against the standard curve of free VP solution.

### Stability assessment of FA-LPHNPs-VP in biological environment

For FBS stability studies, 200 μL of FA-LPHNPs-VP samples were diluted in McCoy's 5A cell medium, with or without FBS. The samples were placed into Pur-A-Lyzer Maxi Dialysis Kit (Molecular weight cut-off 12–14 kDa; Sigma-Aldrich, Cat#PURX12015), which were then submerged in 50 mL centrifuge tubes containing 10 mL of the same medium. These tubes were maintained at room temperature under continuous gentle shaking. At specific time points (1 h, 7 h, 24 h, 48 h, 72 h, 96 h), the fluorescence intensity of VP released from the FA-LPHNPs-VP into the surrounding medium was measured using a fluorescence spectrometer (FluoroMax, Horiba Scientific, Japan). After 96 h, 100 μL of acetonitrile was added to each dialysis device to completely destabilise the nanoparticles, enabling measurement of the total VP fluorescence. The percentage of VP release was calculated using the formula:

$$\text{VP}\;\text{release}\%=V_{n}/V\;\times \;100\%$$

where *V*_n_ is the fluorescence intensity at each time point, and *V* is the fluorescence intensity after complete nanoparticle destabilisation.

For PBS and pH stability studies, 200 μL of FA-LPHNPs-VP samples were incubated in PBS and MES buffer with pH 7.5 (control), 6.0, and 5.0, respectively. These samples were transferred into dialysis devices, which were then placed into 50 mL centrifuge tubes containing 10 mL of the corresponding buffer. The same dialysis procedure and fluorescence measurements described above were followed to assess VP release under different pH conditions.

### Singlet oxygen (^1^O_2_) detection in the solution

400 μL diluted nanoparticle solutions (LPHNPs, FA-LPHNPs-VP) and 6 μL singlet oxygen sensor green (SOSG, Thermo Fisher Scientific, Cat#S36002) (0.5 mM) were mixed. The mixture was then placed in a 48-well plate and irradiated with X-rays (2 Gy, 4 Gy, and 6 Gy), respectively, using a 320 kV cabinet X-Ray Irradiator (X-RAD 320, Precision X-Ray, Inc., Madison, USA). SOSG fluorescence signal was measured using a SpectraMax i3x Multi-Mode Microplate Reader (Molecular Devices, California, USA) at Excitation/emission: 488/525 nm, before and after X-ray exposure. The fluorescence signal increment of SOSG was determined as follow:

$$\text{Intensity}\;\text{increase}\;\text{proportion}\;\text{of}\;\text{SOSG}=\left(F_{2}-F_{1}\right)/F_{1}\;\times \;100\%$$

where F_1_ and F_2_ are SOSG fluorescence intensities before and after X-ray radiation.

### Cell culture

Human colon cancer cell lines (HCT116, Cat# CCL-247; RRID: CVCL_0291; HCT116-Luc2, Cat# CCL-247-LUC2; RRID: CVCL_VU38) were cultured in McCoy's 5A Medium (ATC302007) supplemented with 10% FBS and 1% antibiotic-antimycotic. The normal human colon cell line (CCD841 CoN, Cat# CRL-1790; RRID: CVCL_2871) were cultured Eagle's Minimum Essential Medium (EMEM, ATC302003) with 10% FBS and 1% antibiotic-antimycotic (Thermo Fisher Scientific, Cat#15240062). The cells were cultured at 37 °C in a humidified atmosphere with 5% CO_2_. The cells were regularly checked and confirmed to be negative for mycoplasma contamination and only mycoplasma negative cells were used in all experiments.

### Intracellular ^1^O_2_ and ROS measurements

Intracellular ^1^O_2_ and ROS generation were evaluated using an SOSG probe and 2′,7′-dichlorofluorescein diacetate (DCFH-DA; Sigma-Aldrich, Cat#D6883), respectively. HCT116 cells were treated with 4 Gy X-ray alone, LPHNPs, LPHNPs & 4 Gy, LPHNPs-VP, LPHNPs-VP & 4 Gy, FA-LPHNPs-VP, and FA-LPHNPs-VP & 4 Gy. Following treatments, cells were incubated with SOSG solution (0.5 mM) for 0.5 h or DCFH-DA solution (25 μM in HBSS) for 1 h in darkness. Then, the cells were imaged with the FV3000 confocal laser scanning microscope. A 488 nm laser was applied for exciting DCF and SOSG with the detection wavelength ranging from 500 to 600 nm. The SOSG and DCF signals were analysed by ImageJ (Version: 1.53a), which represented the intracellular ^1^O_2_ and ROS levels produced with different treatments.

### Assessment of cellular uptake

HCT116 and CCD841 cells were seeded at 5.9 × 10^5^ cells and 5.8 × 10^5^ cells respectively onto glass-bottom petri dishes and cultured until 70% confluency. Opti-MEM media mixed with nanoparticles (volume ratio: 1:4) were allowed to incubate for 0.5, 1, 2, and 3 h, respectively. After cells were washed 3 times with DPBS, 1 mL Invitrogen™ Live Cell Imaging Solution (Thermo Fisher Scientific, Cat#A14291DJ) combined with NucBlue™ Live Ready Probes™ Reagent (Hoechst 33342, Thermo Fisher Scientific, Cat#R37605) (5 μg/mL) was added to each Petri dish and imaged under a laser scanning confocal microscope (FV3000; Olympus, Tokyo, Japan). VP and the nuclear dye (Hoechst 33342) were excited at 405 nm wavelength. Images were performed via a UAPON 40 × O340 oil objective lens. Emission signals of VP (680–710 nm) and NucBlue (430–470 nm) were collected. Images were analysed by ImageJ (Version: 1.53a). The corrected total cell fluorescence (CTCF) level was calculated as follows:

CTCF=integratedfluorescencesignal−(selectedarea×meanfluorescenceofbackgroundsignal)

### Nanoparticle toxicity assay

To evaluate the *in vitro* toxicity of these nanoparticles, the MTS assay was carried out. HCT116 (3.4 × 10^5^ mL^−1^) and CCD841 (5.2 × 10^5^ mL^−1^) cells were cultivated in 96-well plates at 37 °C for 24 h. Cells were then cultured with FA-LPHNPs-VP in Opti-MEM solution (Thermo Fisher Scientific, Cat#31985062) for 2 h at 64.35, 128.70, 257.40, and 514.80 μg/mL. After removing the nanoparticle suspension, 100 μL fresh cell medium was added, followed by another 24 h cultivation. The MTS Cell Viability Assay Kit (CellTiter 96® AQueous One Solution Cell Proliferation Assay, Promega, Cat#G3580) was performed as per manufacturer's protocol. SpectraMax i3x Multi-Mode Microplate reader was used to record the absorbance at 490 nm. The HCT116 cell viability was analysed according to the following equation:

$$\text{Viability}\;\left(\%\right)=\left(A_{T}-A_{B}\right)/\left(A_{C}-A_{B}\right)\;\times \;100\%$$

where A_T_ is the treatment group absorbance, A_C_ is cell control group absorbance and A_B_ is cell medium absorbance.

### The RA-PDT treatment on cell viability

The *in vitro* RA-PDT effects were investigated through evaluating cell viability via the MTS and cell viability imaging kits. Briefly, HCT116 cells (3.8 × 10^5^ mL^−1^) were cultured in 96-well plates at 37 °C for one day. Then, cells were exposed to different treatment conditions including cells only, cells & 4 Gy X-ray, LPHNPs, LPHNPs & 4 Gy, LPHNPs-VP, LPHNPs-VP & 4 Gy, FA-LPHNPs-VP, and FA-LPHNPs-VP & 4 Gy. After the treatments, cells were incubated for another 24 h and their viability was measured using the MTS assay as per kit protocol.

For cell viability imaging, HCT116 cells (5.9 × 10^5^ mL^−1^) were seeded into glass-bottom petri dishes and cultivated at 37 °C for 2 days. Then, cells were separated for different treatments: cell only, cells & 4 Gy X-ray, LPHNPs, LPHNPs & 4 Gy, LPHNPs-VP, LPHNPs-VP & 4 Gy, FA-LPHNPs-VP, and FA-LPHNPs-VP & 4 Gy. One day after treatment, the ReadyProbes™ Cell Viability Imaging Kit (Blue/Green) (Thermo Fisher Scientific, Cat#R37609) was employed according to instructions. The blue signal (excitation/emission: 360/460 nm) from all cells' nuclei, and green signal (Excitation/Emission: 504/523 nm) from the dead cells’ nuclei were detected using the FV3000 confocal laser scanning microscope. The percentage of cell viability was analysed according to the following equation:

$$\text{Viability}\;\text{of}\;\text{HCT}116\;\text{cells}\;\left(\%\right)=\left(N_{t}-N_{d}\right)/N_{t}\;\times \;100\%$$

where *N*_*d*_ represents the number of dead cells detected, and *N*_*t*_ means total cell number per group.

### Clonogenic survival assay

HCT116 cells (5.81 × 10^5^ mL^−1^) were seeded in 6-well plates and incubated at 37 °C for 24 h. Cells were then subjected to different treatment conditions: cells + 4 Gy X-ray, LPHNPs + 4 Gy, LPHNPs-VP + 4 Gy, and FA-LPHNPs-VP + 4 Gy. Following treatment, 5000 cells from each group were reseeded per well and incubated for 14 days, with the culture medium refreshed twice a week.

After the incubation period, colonies were fixed with 1 mL of 10% formaldehyde for 30 min and washed twice with DPBS. The cells were then stained with 1 mL of 0.1% crystal violet for 30 min. Excess stains were removed by washing with water, and the plates were allowed to dry at room temperature for 1 h. Colonies containing more than 50 cells were counted manually.

### Apoptosis and necrosis assay

To investigate the cell death pathways after the treatments, we conducted the apoptosis/necrosis assay. In brief, HCT116 cells were stained with Apoptosis/Necrosis Assay Kit (Abcam, Cat#ab176750) according to instructions at 24 h after the treatments, and then imaged under the Olympus FV3000 confocal laser scanning microscope and analysed by flow cytometry (BD LSRFortessa SORP, BD Biosciences, San Jose, CA, USA). Results were analysed through comparison of the number of healthy or apoptotic or necrotic cells to total cell number. This was calculated using the follow equation:

$$\text{Cells}\;\left(\%\right)=A_{h/a/n}/A_{t}\;\times \;100\%$$

where A_h/a/n_ represents healthy or apoptotic or necrotic cell number, and A_t_ is the total cell number per group.

### Cell cycle analysis

To verify if RA-PDT induces HCT116 cell cycle arrest, cell cycle analysis was carried out by using the Luminex Muse® Cell Cycle Kit (Abacus dx, Cat#LUMCH100106). In brief, HCT116 cells (5.5 × 10^5^ mL^−1^) were seeded into 6-well plates and cultivated for 2 days. Subsequently, cells were treated with various treatments: cells only, cells & 4 Gy X-ray, LPHNPs, LPHNPs & 4 Gy, LPHNPs-VP, LPHNPs-VP & 4 Gy, FA-LPHNPs-VP, and FA-LPHNPs-VP & 4 Gy. After 24 h, cells were collected by trypsinisation, washed with cold DPBS, and then suspended in ice-cold 70% ethanol for fixation at −20 °C overnight. Following fixation, HCT116 cells were washed once with cold DPBS then resuspended in 200 μL Muse® Cell Cycle staining reagent and incubated at room temperature for 0.5 h in darkness. Cell cycle distribution was assessed using the Muse® Cell Analyser (Luminex Corporation, Texas, USA).

### Ethics

All animal experiments were reviewed and approved by the UNSW Animal Care and Ethics Committee (ethics approval 20/95B, 21/39B, 21/77B, 22/30B, and 23/48B). NOD/SCID (6–8-week-old) and BALB/c nude mice (6–8-week-old) were provided by Animal Services from the Animal Resources Centre (ARC, Perth, WA). All procedures were conducted in full compliance with the ARRIVE guidelines and relevant ethical standards.

### Mice care

Mice were housed in specific pathogen-free conditions at 22 °C with a 12-h light/dark cycle, kept in standard ventilated cages with a maximum of five mice per cage in UNSW. All mice acclimated for one week following arrival at the UNSW animal facility. They were provided with sterile pellet food and water ad libitum, and their well-being was monitored regularly. To minimise clustering effects, mice were randomly assigned to experimental groups, with littermates distributed across different groups. The experimental period was limited to 8 weeks. All animals were humanely euthanised in accordance with institutional guidelines using CO_2_ inhalation followed by cervical dislocation when the endpoint is reached.

Female NOD/SCID (6–8-week-old) and BALB/c nude mice (6–8-week-old) are applied for this study. Female mice are preferred due to their hormonal stability, behavioural consistency and more predictable immune responses, which contribute to reduced variability and more reliable results in tumour growth and treatment efficacy studies.

### Orthotopic rectal cancer model

An orthotropic rectal cancer mouse model was established by using intra-rectal tumour cell injection method.^34^ In brief, 6–8-week-old female NOD/SCID mice were fasted for 6 h prior to injections. They were anaesthetised with inhalation anaesthetics for the injection. Lubricated blunt-tip forceps were used to dilate the anal canal, exposing the distal anal and rectal mucosa. 4 × 10^5^ HCT-116-Luc2 cells suspended in 10 μL PBS and 10 μL Matrigel were inoculated in the distal posterior rectal submucosa, 1–2 mm above the anal canal using a 30-gauge needle (Terumo, Tokyo, Japan).

Tumour formation and growth over time were monitored once a week by using the IVIS Spectrum CT imaging system (PerkinElmer, Waltham, US). Mice were intraperitoneally (IP) injected with 150 mg/kg of Pierce™ D-Luciferin Monopotassium Salt (Thermo Fisher Scientific, Cat#88294) prior to imaging. Imaging data was analysed with Living Image® 4.5.2 software. Tumour growth was also monitored with ultrasound imaging (Vevo 3100, FUJIFILM VisualSonics Inc, Toronto, Canada). Briefly, mice were anaesthetised and hair in the region of interest was removed by hair removal cream. The ultrasound transducer (MX400) was placed over the region of interest and a 3D acquisition of the tumour was performed. Volumetric calculations of the tumour size were made using the VevoLab 3.2.5 software.

### *In vivo* nanoparticle safety assessment

Acute toxicity of FA-LPHNPs-VP nanoparticles was assessed with single intravenous (IV) injection at doses of 12.5 mg/kg, 25 mg/kg, and 50 mg/kg (n = 3 per group). Mice were closely monitored for 72 h post-injection for any adverse effects including changes in body weight, appearance, behavioural patterns and to detect any signs of pain, illness or distress. Subacute toxicity of nanoparticles was assessed with multiple IV injections of FA-LPHNPs-VP at 12.5 mg/kg, 25 mg/kg, and 50 mg/kg (n = 3 mice per group) given on days 0, 2, 4, and 6. Mice were monitored daily for 7 days to assess any clinical signs of toxicity. After 7 days, mice were euthanised and the organs (heart, liver, spleen, lung, and kidneys) were collected for histological analysis. The tissues were fixed in 10% neutral buffered formalin for one day, embedded in paraffin and cut into 4 μm sections. Slides were deparaffinised and stained with haematoxylin and eosin (H&E). All slides were scanned with the Olympus SLIDEVIEWTM VS200 research slide scanner (Olympus, Japan), which were performed by Katharina Gaus Light Microscopy Facility (The University of New South Wales, Sydney, Australia). Slide images were analysed by QuPath-0.3.2 software.

### Haematological analysis after repeated nanoparticle dosing

Mice were randomly divided into four groups (n = 3 per group) and IV injected with (1) 200 μL PBS and (2–4) 200 μL FA-LPHNPs-VP nanoparticles at 12.5, 25, and 50 mg/kg. The injection was conducted on days 0, 2, 4, and 6. At day 48, mice were euthanised and 500–750 μL blood was collected per mouse using the cardiac puncture technique with a 25-gauge needle. Blood was collected into K3 EDTA tubes (Icon Supplies Pty Ltd, Cat#450474), allowing analysis of whole blood with the Sysmex XN-V haematology analyser, which was performed by the Veterinary Pathology Diagnostic Services (The University of Sydney, Sydney, Australia).

### Innate immune system assessment after repeated nanoparticle dosing

To assess the immunotoxicity of repeated FA-LPHNPs-VP dosing, the Bio-Plex cytokine assay system was utilised to screen the cytokines TNF alpha, INF gamma and IL-6. Briefly, mice were administrated with nanoparticle suspension at different concentrations every other day as described above (Haematological analysis after repeated nanoparticle dosing). After the final injection on day 6, mice (n = 3/group/timepoint) were euthanised at 30 min, 2 h, 5 h, 24 h, and 7 days, followed by terminal blood collection performed via the aforementioned cardiac puncture technique. Sodium citrate tubes were used for collection of blood samples, allowing isolation of blood plasma by centrifuging at 1000×*g* for 10 min. Afterwards, cytokine levels in the blood plasma were analysed using the LEGEND MAX™ Mouse TNF-α (BioLegend, Cat#430907), IFN-γ (BioLegend, Cat#430807) or IL-6 (BioLegend, Cat#431307) ELISA Kit based on manufacturer's specifications.

### Assessment of nanoparticle biodistribution

The biodistribution of nanoparticles in various organs and tumour tissue was evaluated over a 24 h period by analysing nanoparticle accumulation. Orthotopic tumour-bearing mice were intravenously injected with 50 mg/kg of nanoparticles. Three mice per group were used for accumulation assessment at each time point (1 h, 4 h, 6 h, and 24 h post-injection). The mice were euthanised, and their organs, including the heart, liver, spleen, lung, kidneys, and rectal tumour, were excised and examined using fluorescence imaging via the IVIS Spectrum CT imaging system (*Ex* 425 nm, *Em* 690 nm). The concentration of VP in frozen tumour tissues at collected at different time point were measured via spectrofluorometer (FluoroMax-4 HORIBA Scientific Co, Kyoto, Japan) (Excitation/Emission: 425 nm/690 nm) after extraction into acetonitrile. Statistical analysis of the data was performed using two-way ANOVA to evaluate differences in biodistribution across time points and organ types.

### *In vivo* anti-tumour evaluation in an orthotopic mouse model

When the bioluminescence signal from tumours reached approximately 4–6 × 10^10^ photons/s, which was between day 14 and day 21 after HCT-116-Luc2 cells implantation, mice were randomly divided into four groups and treated as follows: (1) PBS, (2) X-ray alone, (3) FA-LPHNPs-VP alone, and (4) FA-LPHNPs-VP & X-ray. 200 μL PBS or 200 μL FA-LPHNPs-VP suspension in PBS (50 mg/kg) was IV administered in mice of group (1), (3), & (4), respectively, followed by X-ray radiation at 4 h post-injection for group (2) and (4). Tumour growth was detected using bioluminescence imaging through weekly IVIS SpectrumCT imaging. At 27 days post-treatment, mice were euthanised, and tumour tissues were collected. The tumour volume (V) was calculated as V = π/6 × length × width × height.^35^, ^36^, ^37^

### Development of a mouse model bearing lymph node metastasis

The required model was established by injecting HCT116-Luc2 cells into the footpad. In brief, 6–8-week-old female BALB/c nude mice (n = 10 mice per group) were anaesthetised with inhalation anaesthetics and then 4 × 10^6^ HCT116-Luc2 cells suspended in 40 μL PBS were inoculated in right footpad using a 27-gauge needle (Terumo, Tokyo, Japan). Tumour growth over time was monitored once a week by using a IVIS Spectrum CT imaging system (PerkinElmer, Waltham, US). Mice were IP injected with 150 mg/kg of D-Luciferin prior to imaging. Imaging data was analysed with Living Image® 4.5.2 software.

### Assessment of FA-LPHNPs-VP accumulation in metastatic lymph node tissues

Firstly, we compared the FA-LPHNPs-VP injection methods by assessing VP fluorescence intensity in lymph node tissues at 4 h post injection. Specifically, mice were randomly divided into three groups: (1) IV injection of 50 mg/kg FA-LPHNPs-VP in 200 μL PBS; (2) IP injection of 50 mg/kg FA-LPHNPs-VP in 200 μL PBS; and (3) hock injection of 12.5 mg/kg FA-LPHNPs-VP in 50 μL PBS. At 4 h post-injection, mice were euthanised, and lymph node tissues were collected. Then collected lymph node tissues were imaged under a IVIS Spectrum CT imaging system (*Ex* 425 nm, *Em* 690 nm).

After confirming the injection method, we then examined the accumulation profile of FA-LPHNPs-VP at different timepoints. Briefly, mice (n = 12 mice) were randomly divided into four groups: (1) IP injection of 200 μL PBS, n = 3 mice and (2–4) IP injection of 50 mg/kg FA-LPHNPs-VP in 200 μL. At 6 h, 12 h, and 24 h (n = 3 mice per timepoint) after injection the mice were euthanised. The collected lymph node tissues were imaged using a IVIS Spectrum CT imaging system (*Ex* 425 nm, *Em* 690 nm).

### *In vivo* anti-tumour evaluation for lymph nodes metastasis

On day 10 after HCT116-Luc2 cells implantation, 40 mice were randomly divided into four groups (10 mice per group) and treated as follows: (1) PBS, (2) X-ray alone, (3) FA-LPHNPs-VP (50 mg/kg) alone, and (4) RA-PDT. Tumour growth was monitored weekly using a IVIS Spectrum CT imaging system. On day 18 post-treatment, mice were euthanised, and both tumour and lymph node tissues were collected. The tumour volume (V) was calculated as V = 0.5 × length × width × height. Primary tumour tissues were assessed by histological analysis, while lymph node tissues were assessed by IHC analysis.

### Histological and IHC analysis on primary tumour and lymph node tissues

Primary tumours were placed into 10% neutral buffered formalin for fixation one day before being embedded in paraffin blocks. The blocks were then cut into 4 μm sections, deparaffinised and stained with H&E. To further evaluate the mitotic activity in primary tumours and lymph node tissues, Ki-67 expression level was assessed using the Ki-67 Recombinant Rabbit Monoclonal Antibody (Thermo Fisher Scientific Cat# MA5-14520, RRID: AB_10979488; 1:2000). Heat-induced antigen retrieval in pH 6.0 citrate buffer at 110 °C for 5 min was performed. The anti-rabbit Poly-HRP-IgG and 3,3′-diaminobenzidine (DAB) in BOND Polymer Refine Detection (Leica Biosystems Cat# DS9800, RRID: AB_2891238) were applied for IHC staining detection. Cytokeratin 19 staining was conducted using Cytokeratin 19 Rabbit monoclonal antibody (Abcam Cat# ab76539, RRID: AB_1523469; 1:1000).

### Sample slide imaging and data analysis

H&E, Ki-67-stained and Cytokeratin 19-stained slides were scanned by using an Olympus SLIDEVIEW™ VS200 research slide scanner (Olympus, Japan) at 40×. For differentiation of live tumour cells versus (vs.) necrotic areas, images stained by H&E were partitioned via thresholding in QuPath-0.3.2 software, which can classify cells into groups based on colour. The purple areas in tumour tissues indicates haematoxylin staining of cellular nuclei, classified as live tumour. The cytoplasm of nuclei-free dead cells stained by eosin shows a prevailing pink colour. The total area of live and necrotic cells was identified as proportion of the entire sample section area. In addition, the percentage of Ki-67 and Cytokeratin 19 positive cells in the slides were assessed with thresholds of 1+, 2+, and 3+ to detect different intensities of Ki-67 and Cytokeratin 19 positive cells using the QuPath-0.3.2 software.

### Statistics

All statistical analyses and graphs were performed by GraphPad prism version 9.2.0 for Windows software. The results data in this study are presented as means with 95% confidence intervals (95% CI) and mean ± standard deviation (SD)/standard error of the mean (SEM) as indicated, n ≥ 3. Normality of all variable data was assessed via Shapiro–Wilk test and generated Q–Q plots. Homogeneity of variance was confirmed by examining the homoscedasticity of residual plots. Differences between groups were analysed by two-way analysis of variance (ANOVA) with Tukey's multiple comparison post hoc test. Two-tailed unpaired Student's t-test was performed for unmatched groups. A p-value below 0.05 was considered to indicate a statistical significance. A priori statistical power analysis for *in vivo* RA-PDT therapeutic effect on orthotopic rectal cancer and lymph node metastasis tumour was performed, which estimated that ten animals per groups were sufficient to obtain a power of 0.80 with a significance p < 0.05.

### Role of funders

The funders of this study had no role in the study design, data collection, data analyses, interpretation of results, or manuscript drafting.

## Results

### Preparation and characterisation of lipid-polymer hybrid nanoparticles

**Fig. 2:**
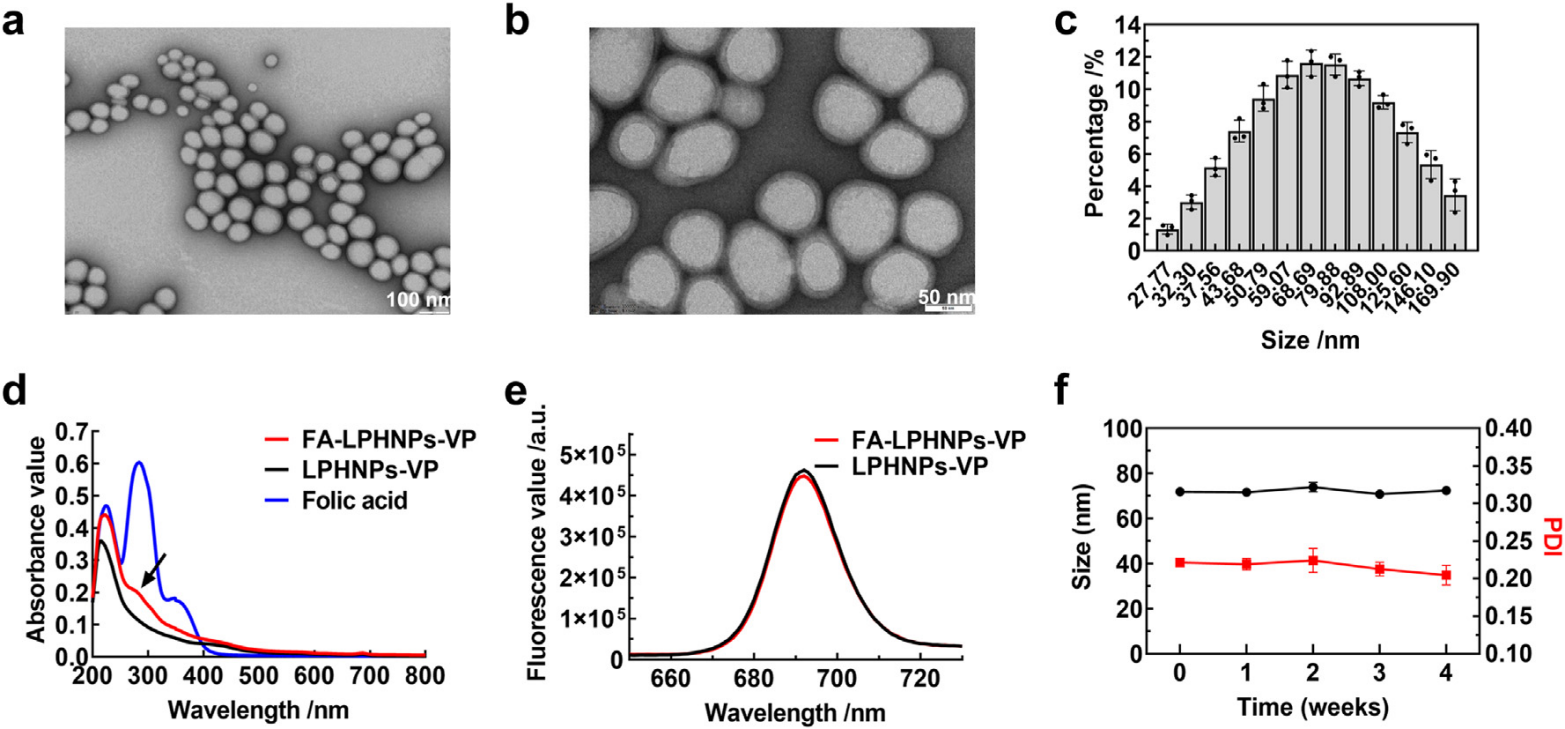
**Characterisation of polymer-lipid hybrid nanocarriers prepared in our study.** TEM images of FA-LPHNPs-VP at (a) 100,000× and (b) 300,000× magnification (scale bar, 100 and 50 nm, respectively); (c) Size distribution of FA-LPHNPs-VP/intensity; (d) The absorbance spectra of LPHNPs-VP, Folic acid molecules and FA-LPHNPs-VP, the arrow indicates the peak at 282 nm for FA-LPHNPs-VP; (e) Fluorescence spectra of LPHNPs-VP and FA-LPHNPs-VP; (f) Long-term stability study of FA-LPHNPs-VP by checking particle size (black line) and PDI (red line) in water which were monitored for 4 weeks at 4 °C (Two-way ANOVA with Tukey's multiple comparison post hoc test). Data is described as mean ± SD of three independent experiments.

Size and morphology analysis shows that the FA-LPHNPs-VP nanoparticles had spherical shapes of around 70 nm with a distinct core-shell structure visible under high magnification (Fig. 2a and b). The average hydrodynamic diameters of FA-LPHNPs-VP was 71.89 nm (95% CI: 70.72 nm, 73.06 nm) via Dynamic Light Scattering (DLS) measurement (Fig. 2c), comparable with the calculated diameters from the acquired TEM images. In addition, the zeta potential (indicative of the surface charge) of three nanoparticles (LPHNPs, LPHNPs-VP, and FA-LPHNPs-VP), via electrophoretic light scattering (ELS) measurement, showed a similar negative charge (Table 1). The low and similar polydispersity indices (PDI) confirm that these nanoparticles are monodisperse. To validate the successful modification of FA in FA-LPHNPs-VP, we assessed and compared the absorbance spectra of LPHNPs-VP, FA molecules and FA-LPHNPs-VP (Fig. 2d). A peak at around 282 nm for FA, was also observed in FA-LPHNPs-VP, conversely, this peak was not detected in LPHNPs-VP (Fig. 2d). These findings confirm the modification of FA-LPHNPs-VP with FA molecule. The incorporation efficiency of the DSPE-PEG-FA polymer component within the FA-LPHNPs-VP formulation was approximately 96.78% (95% CI: 93.38%, 100.18%), as calculated using a standard curve (Fig. S1). In addition, in the FTIR spectra of DSPE-PEG-FA (Fig. S2a) and FA-LPHNPs-VP (Fig. S2b), peaks appeared at 1605 cm^−1^ and 950 cm^−1^ were observed. These peaks were absent in the spectra of LPHNP-VP (Fig. S2c) and LPHNPs (Fig. S2d), which further confirmed the success modification of our nanoparticles with FA.^38^^,^^39^

**Table 1:** The average diameters, zeta potential and PDI of LPHNPs measured by DLS.

Nanoparticles	Zeta (95% CI; mV)	Size (95% CI; d. nm)	PDI (95% CI)	EE% (95% CI)
LPHNPs	−31.68 (−32.68, −30.67)	66.70 (66.07, 67.40)	0.25 (0.24, 0.25)	–
LPHNPs-VP	−29.74 (−31.44, −28.05)	69.36 (66.73, 72.66)	0.22 (0.21, 0.22)	86.11 (85.28, 86.94)
FA-LPHNPs-VP	−29.81 (−30.50, −29.12)	71.89 (70.72, 73.06)	0.21 (0.18, 0.23)	92.31 (91.10, 93.51)

The VP loading was evaluated by assessing the fluorescence intensity of the nanoparticles, and the results show that both LPHNPs-VP and FA-LPHNPs-VP were fluorescing at 690 nm with similar intensity (Fig. 2e), indicating a comparable concentration of VP encapsulation in LPHNPs-VP and FA-LPHNPs-VP. The EE% of VP was also calculated, with high EE% of VP in LPHNPs-VP and FA-LPHNPs-VP being observed (86.11% (95% CI: 85.28%, 86.94%) and 92.31% (95% CI: 91.10%, 93.51%), respectively; Table 1).

The colloid stability of FA-LPHNPs-VP was examined by checking size change every 7 days over a period of 4 weeks. As shown in Fig. 2f, there were no statistically significant changes (p > 0.05; Two-way ANOVA with Tukey's multiple comparison post hoc test) in nanoparticle size and PDI (approximately 70 nm with a PDI of ∼0.2 over 4 weeks), indicating the FA-LPHNPs-VP have good stability for 4 weeks without aggregation and precipitation in water. The stability of FA-LPHNPs-VP in a biological environment was further assessed by evaluating VP release using the dialysis technique. As shown in Fig. S3a, FA-LPHNPs-VP exhibited comparable VP release profiles in cell media with and without FBS. Additionally, Fig. S3b and c demonstrates that the nanoparticles-maintained stability across pH values ranging from 5.0 to 7.5 and in PBS buffer, with less than 3% VP released over 96 h. These findings indicate that FA-LPHNPs-VP remain stable under the experimental conditions, with negligible drug leakage from the carriers.

### The effect of RA-PDT treatment on cell viability

**Fig. 3:**
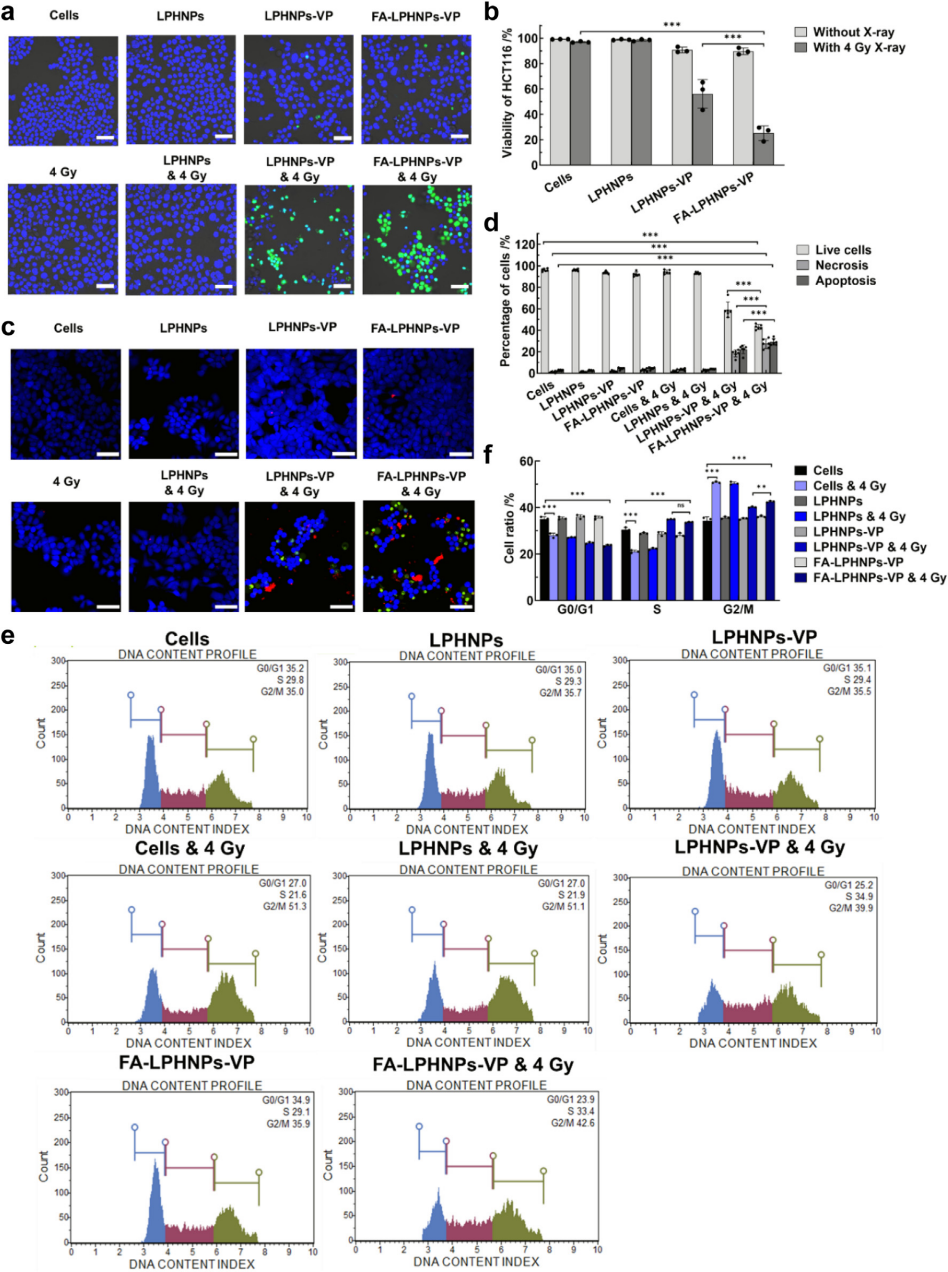
***In vitro* RA-PDT effect evaluation.** (a) Representative confocal microscopy images of HCT116 cells at 24 h after different treatments. Scale bar is 50 μm. The green signals indicate dead cells; (b) Quantitative analysis of viable cell percentage based on live/dead cell assay (Two-way ANOVA with Tukey's multiple comparison post hoc test); (c) The confocal microscopy images of healthy, apoptotic and necrotic cells at 24 h after different treatments. The blue signal indicated healthy cells, red signal suggested apoptotic cells and green colour represented necrotic cells. Scale bar, 50 μm; (d) Quantitative analysis of cell population changes in apoptosis/necrosis levels as depicted in Fig. 3c (Two-way ANOVA with Tukey's multiple comparison post hoc test); (e) HCT116 cell cycle distribution at 24 h after treatments; (f) The proportion of cell population in G0/G1, S, and G2/M phases at 24 h (Two-way ANOVA with Tukey's multiple comparison post hoc test). Mean ± SD of three independent experiments where each dot represents the mean of 2–3 replicates for a–d and three independent experiments for e and f; ∗∗p < 0.01; ∗∗∗p < 0.005.

After confirmation of *in vitro* targeting ability, intracellular ^1^O_2_ and ROS generation and dark toxicity of FA-LPHNPs-VP (Figs. S4, S5, and S6), the *in vitro* RA-PDT effect in HCT116 cells was evaluated via a live/dead cell and MTS assays. As shown in Fig. 3a and b and Fig. S7, cells treated with LPHNPs alone and X-ray alone did not exhibit reduced viability compared with the control group. Cells treated with LPHNPs-VP and FA-LPHNPs-VP experienced minor reduction in cell viability, at 91% (95% CI: 88.22%, 93.19%) and 90% (95% CI: 86.80%, 92.59%), respectively. However, when these cells were treated with RA-PDT, the viability was considerably (p < 0.005; Two-way ANOVA with Tukey's multiple comparison post hoc test) reduced to 56.09% (using LPHNPs-VP; 95% CI: 51.57%, 60.60%) and 25.20% (using FA-LPHNPs-VP; 95% CI: 18.66%, 31.75%), respectively. This statistically significant decrease confirms that FA-LPHNPs-VP & 4 Gy showed a higher level of cell killing than LPHNPs-VP & 4 Gy, highlighting its enhanced therapeutic potential. This could be attributed to enhanced cellular uptake of FA-modified nanoparticles in HCT116 cells. When these findings were further verified with the MTS assay, a similar trend was observed (Fig. S8). In addition, the clonogenic survival assay was conducted to further evaluate the therapeutic effects of RA-PDT. As shown in Fig. S9, following 4 Gy X-ray irradiation, the number of colonies in the control and LPHNPs-treated groups remained comparable, with 95.33 (95% CI: 88.70, 101.96) and 91.00 (95% CI: 88.00, 93.99) colonies, respectively. In contrast, the LPHNPs-VP-treated group exhibited a marked reduction, with only 0.67 (95% CI: 0.01, 1.32; p < 0.005; Two-way ANOVA with Tukey's multiple comparison post hoc test) colonies. Notably, no cell colonies were observed in the FA-LPHNPs-VP & 4 Gy-treated group, indicating that this treatment effectively inhibited HCT116 cell growth. Taken together, these results indicate that FA-LPHNPs-VP combined with X-ray (4 Gy) resulted in RA-PDT effects which considerably reduced the cell viability. This formulation was selected for further evaluation in a mouse model.

### Evaluation of cell death pathways: apoptosis/necrosis assay

To investigate the cell death pathways in the treatment conditions, apoptosis/necrosis assays were carried out via both confocal imaging (Fig. 3c and d) and flow cytometry (Fig. S10). An observation similar to the cell viability evaluation was noted from the fluorescence signals and their quantitative analysis (Fig. 3c and d). The very low percentage of apoptotic/necrotic cell populations was detected in LPHNPs-VP (6%) and FA-LPHNPs-VP (7%) treated groups, compared with the control group (4%), suggesting that nanoparticles alone showed negligible cytotoxicity and anti-tumour effect towards HCT116 cells. However, in combination with X-ray radiation, notable apoptosis/necrosis percentage increases were observed in LPHNPs-VP and FA-LPHNPs-VP treated groups, compared with control group (p < 0.005; Two-way ANOVA with Tukey's multiple comparison post hoc test). The cell group treated with FA-LPHNPs-VP & 4 Gy demonstrated the highest percentage of apoptosis/necrosis percentage, of 29.29% (95% CI: 26.83%, 31.75%) and 27.61% (95% CI: 24.10%, 31.13%), respectively (Fig. 3d). Correspondingly, the lowest percentage of healthy cells was also observed in the FA-LPHNPs-VP & 4 Gy group (43.09% (95% CI: 41.27%, 44.92%)). Overall, these findings suggest that in our study RA-PDT triggered HCT116 cell death through the apoptosis and necrosis pathways.

### Investigation on apoptotic death process: cell cycle analysis

We further performed a cell cycle assay to check if RA-PDT-induced apoptosis was regulated by cell cycle progression. As shown in Fig. 3e and f, in the LPHNPs, LPHNPs-VP, and FA-LPHNPs-VP groups, the cells displayed a similar phase distribution compared with the control at 24 h after treatment, indicating that the nanoparticles alone have no statistically significant effect on HCT116 cell cycle progression (p > 0.05; Two-way ANOVA with Tukey's multiple comparison post hoc test). However, in the groups treated with LPHNPs-VP & 4 Gy and in the FA-LPHNPs-VP & 4 Gy groups, higher cell populations in S and G2/M phases were observed, while the cell population in the G0/G1 phase was reduced considerably compared with the control. Moreover, the FA-LPHNPs-VP & 4 Gy treatment led to a higher cell percentage in the G2/M phase (42.58% (95% CI: 42.20%, 42.96%) vs. 40.17% (95% CI: 39.87%, 40.46%), p < 0.005; Two-way ANOVA with Tukey's multiple comparison post hoc test) and slightly fewer cells in the S-phase (33.69% (95% CI: 33.44%, 33.94%) vs. 34.97% (95% CI: 34.85%, 35.09%), p < 0.005; Two-way ANOVA with Tukey's multiple comparison post hoc test) compared with the LPHNPs-VP & 4 Gy group.

Interestingly, compared with the control group, X-ray treated groups (4 Gy X-ray alone and LPHNPs & 4 Gy) led to markedly increased fraction of cells in the G2/M phase, from 34.33% (95% CI: 32.44%, 36.23%) to 50.89% (95% CI: 50.48%, 51.31%) and 50.48% (95% CI: 49.80%, 51.16%), respectively, but a decreased population in G0/G1 (from 35.05% (95% CI: 33.89%, 36.21%) to 28.02% (95% CI: 26.95%, 29.09%) and 27.18% (95% CI: 26.97%, 27.38%), respectively) and S phases (from 30.53% (95% CI: 29.56%, 31.51%) to 21.07% (95% CI: 20.40%, 21.74%) and 22.30% (95% CI: 21.84%, 22.76%), respectively) at 24 h (p < 0.005; Two-way ANOVA with Tukey's multiple comparison post hoc test). This suggests cell DNA damage repair after X-ray radiation, leading to a temporary G2/M phase arrest.^40^ This is further supported by the observed increase in the G0/G1 phase population and the decrease in the G2/M phase population at 48 h in X-ray-treated groups, as shown in Fig. S11. Collectively, these results indicate that our RA-PDT strategy effectively inhibited the proliferation of HCT116 cells through the S- and G2/M phase arrest, ultimately resulting in HCT116 cell apoptosis.

### *In vivo* RA-PDT therapeutic effect in an orthotopic model bearing a primary tumour

**Fig. 4:**
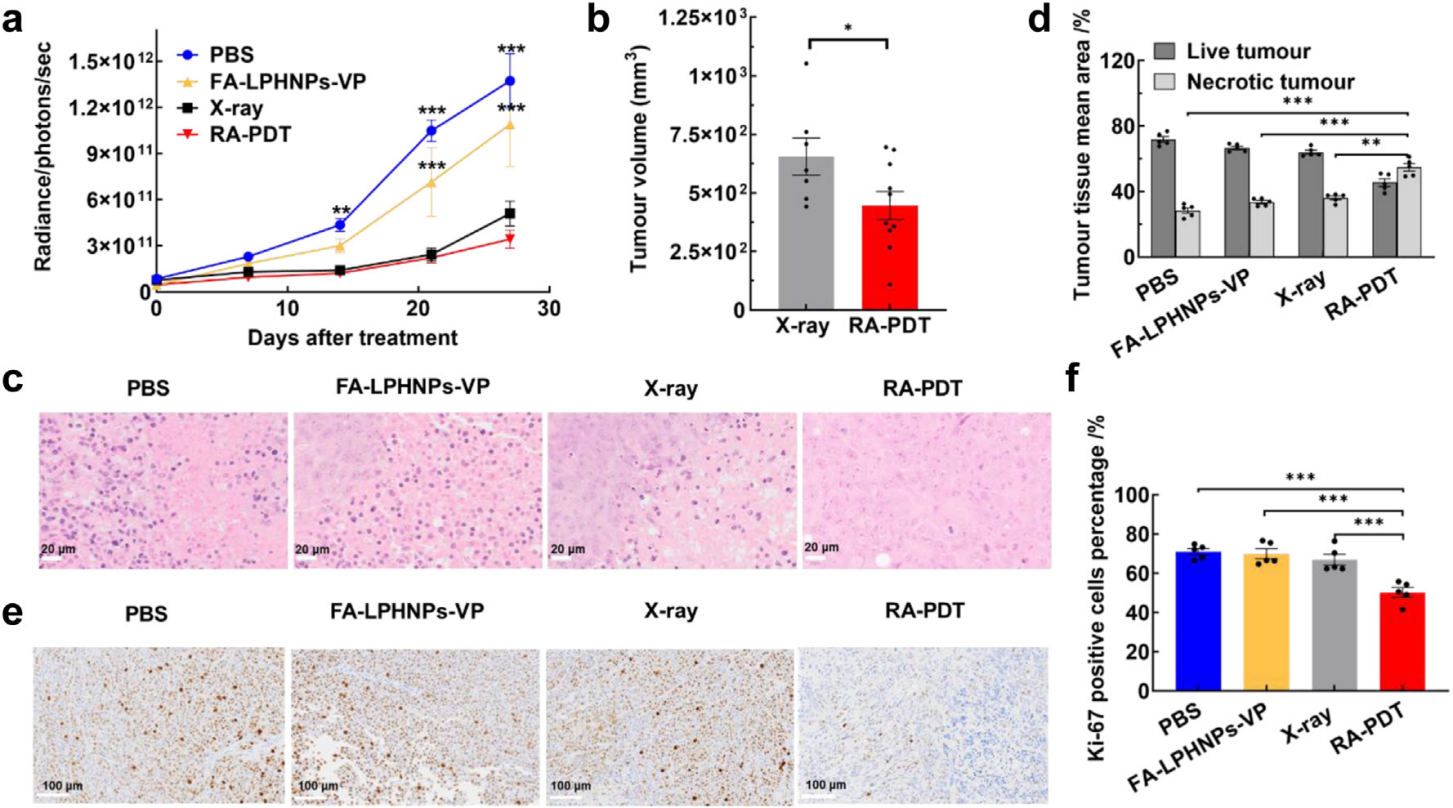
***In vivo* antitumour activity of RA-PDT in an orthotopic mouse model.** (a) Changes in bioluminescence signal from tumours measured by IVIS SpectrumCT imaging within 27 days post treatment as indicated. (Two-way ANOVA with Tukey's multiple comparison post hoc test, n = 7–10 mice per group); (b) The mean tumour volumes for X-ray and RA-PDT groups at day 27 post-treatment. FA-LPHNPs-VP dose was 50 mg/kg and X-ray irradiation dose was 4 Gy, (Two-tailed unpaired Student's t-test, n = 7–10 mice per group); (c) Representative H&E-stained images of tumour tissue sections at endpoint. Scale bar is 20 μm; (d) The percentage of live and necrotic tumour tissue present as detected by H&E-staining in the studied groups (Two-way ANOVA with Tukey's multiple comparison post hoc test, n = 5 mice per group); (e) Representative stained-sections of control and treated tumours for Ki-67 at the endpoint. Scale bar is 100 μm; (f) The percentage of Ki-67 positive cells present as detected by immunohistochemistry in tumour tissues (Two-way ANOVA with Tukey's multiple comparison post hoc test, n = 5 mice per group). Error bars show standard error of mean, ∗p < 0.05; ∗∗p < 0.01; ∗∗∗p < 0.005.

After having confirmed *in vitro* RA-PDT efficacy and *in vivo* safety profile of FA-LPHNPs-VP, we evaluated the RA-PDT effect in an orthotopic rectal tumour model (Figs. S12, S13, and S14). As shown in Fig. 4a, the RA-PDT group shows a notable ability to effectively control tumour growth at 27 days post-treatment, compared with other treatment conditions (RA-PDT vs. FA-LPHNPs-VP and RA-PDT vs. PBS, p < 0.005; Two-way ANOVA with Tukey's multiple comparison post hoc test). Despite RA-PDT group did not show statistically significant differences in the bioluminescence signal, compared with X-ray treated group (RA-PDT vs. X-ray, p = 0.2641; Two-way ANOVA with Tukey's multiple comparison post hoc test), the tumour volume calculated from collected tumours demonstrated a statistically significant difference between these two groups (p < 0.05, Fig. 4b; Two-tailed unpaired Student's t-test). This discrepancy may be attributed to the high variability in bioluminescence signals in larger tumours (>100 mm^3^), as indicated by the weak correlation between bioluminescence signal and tumour size measured by a calliper (Fig. S15, R^2^ = 0.2967).^41^ We further carried out a quantitative analysis of the relative amount of live and dead tissue in the tumours based on H&E-stained images (Fig. 4c). The group treated with RA-PDT exhibited about 55% (95% CI: 50.09%, 59.23%) of necrotic tumour tissue, notably higher than other control groups (RA-PDT vs. PBS; RA-PDT vs. FA-LPHNPs-VP and RA-PDT vs. X-ray, p < 0.005, Fig. 4d; Two-way ANOVA with Tukey's multiple comparison post hoc test). These data indicate that RA-PDT effect led to higher tumour necrosis against tumour progression. Furthermore, we carried out immunohistochemical analysis of mitotic activity in tumour tissues to shed light on the possible mechanisms of tumour inhibition in the RA-PDT group. As shown in Fig. 4e and f, RA-PDT induced a statistically significant decrease in mitotic activity (50% (95% CI: 43.91%, 56.60%) Ki-67 positive cells) in the tumour tissue, when compared with other treatments (71% (95% CI: 66.95%, 74.96%) in PBS, 70% (95% CI: 63.58%, 76.37%) in FA-LPHNPs-VP, and 67% (95% CI: 59.86%, 74.02%) in X-ray, p < 0.005; Two-way ANOVA with Tukey's multiple comparison post hoc test). This indicates that the RA-PDT technique (FA-LPHNPs-VP & 4 Gy) exhibited a more pronounced cytostatic effect on tumours than individual techniques (nanoparticle alone and X-ray alone). Collectively, the results displayed that RA-PDT inhibited the tumour growth by inhibiting cell doubling and increasing cell death.

### *In vivo* RA-PDT therapeutic effect on lymph node tumour

**Fig. 5:**
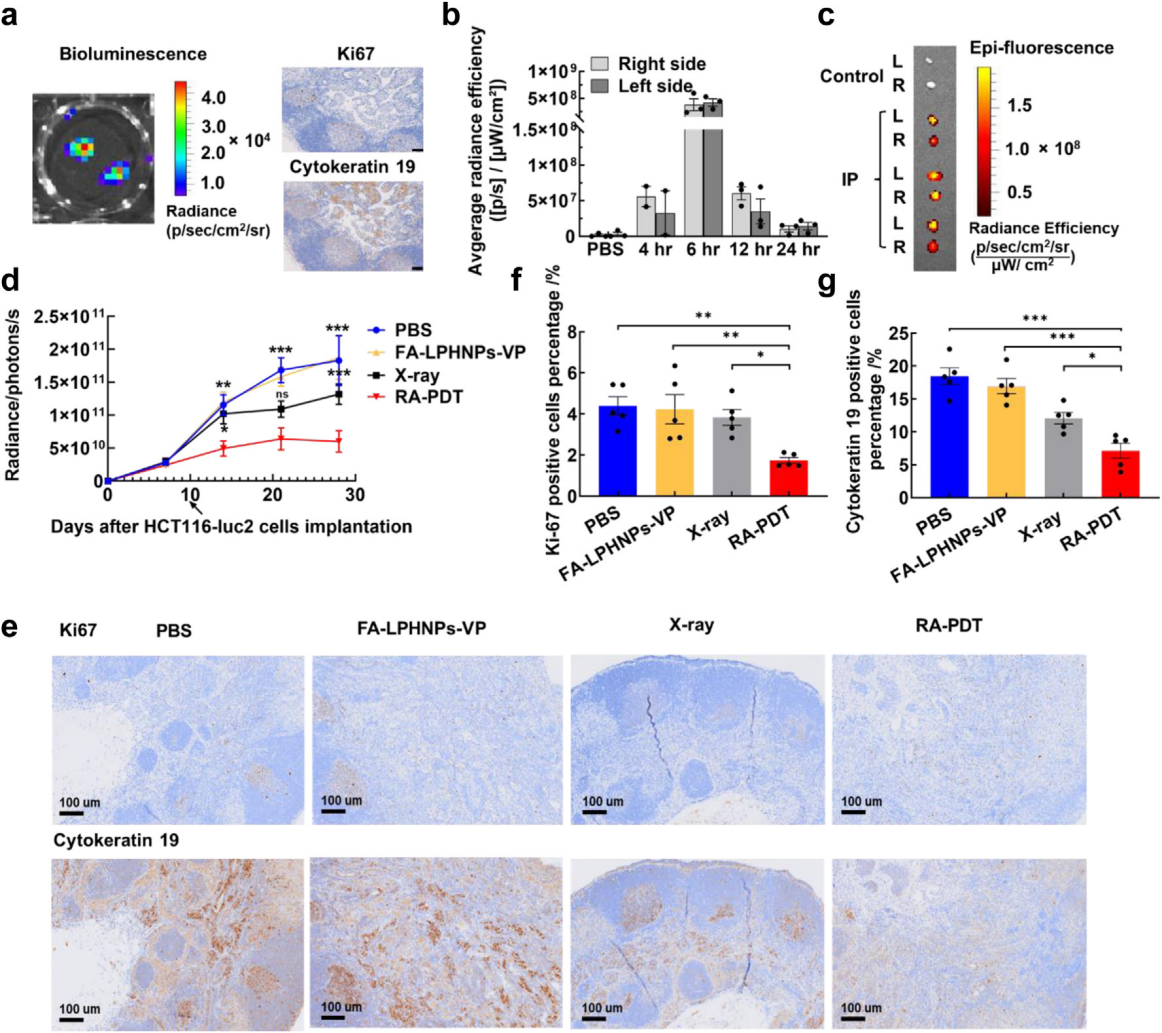
***In vivo* anti-metastasis effect of RA-PDT in a mouse model bearing lymph node metastasis.** (a) Bioluminescence images of lymph node tissues collected at Day 21 after HCT116-Luc2 cell injection into the footpad; Immunohistochemical analysis of lymph node samples with cytokeratin (metastatic marker) and Ki67 (cell proliferation marker) staining and identified the presence of metastatic tumour foci; (b) The quantitative data analysis of VP fluorescence signal in inguinal lymph node tissues at 4, 6, 12, and 24 h post IP injection of FA-LPHNPs-VP (Two-way ANOVA with Tukey's multiple comparison post hoc test, n = 2–3 mice per group); (c) *Ex vivo* fluorescence images of inguinal lymph node tissues collected from both right and left sides of the primary tumour at 6 h post IP injection of FA-LPHNPs-VP; (d) Changes in bioluminescence signals from primary tumours after different treatments within 27 days, the arrow indicates treatment day. (Two-way ANOVA with Tukey's multiple comparison post hoc test, n = 10 mice per group); (e) Representative-stained sections of inguinal lymph node tissues for Ki-67 and Cytokeratin 19 at the endpoint. Scale bar is 100 μm; The percentage of (f) Ki-67 and (g) Cytokeratin 19 positive cells present in inguinal lymph node tissues (Two-way ANOVA with Tukey's multiple comparison post hoc test, n = 5 mice per group); Error bars show standard error of mean, ∗p < 0.05; ∗∗p < 0.01; ∗∗∗p < 0.005.

To evaluate the RA-PDT efficacy against lymph node-positive tumour, the required model was developed by subcutaneously injecting HCT-116-Luc2 cells into the right footpad of mice. The tumour growth was monitored by bioluminescence intensity of cancer cells via bioluminescence imaging. Although it was challenging to observe *in vivo* bioluminescence signals from lymph node areas due to strong signals originating from the injected footpad (Fig. S16), the *ex vivo* signals from inguinal lymph node tissues were clearly detected, suggesting the presence of cancer cells in lymph nodes (Fig. 5a). Furthermore, increased epithelial cancer cells (cytokeratin 19-positive cells, Fig. 5a) and cell proliferation (Ki67, proliferation index, Fig. 5a) were observed from the lymph node tissue, providing the strong evidence of development of metastatic tumour in lymph node tissues.

We further examined the ability of FA-LPHNPs-VP to target cancer cells in lymph nodes by using various administration methods (IV, IP, and hock injections, Figs. S17 and S18a). As shown in Fig. S18b, the IP injection group displayed the highest VP fluorescence signal level in the lymph node tissues, as compared to other administration groups, suggesting a potential transport pathway in which nanoparticles exit the peritoneal cavity via stomata, particularly in milky spots, subsequently enter lymphatic lacunae, and drain into the lymphatic system, facilitating their accumulation in lymph nodes.^42^ The accumulation profile of nanoparticles in metastatic lymph node tissues was also assessed. The strongest VP fluorescence signal from FA-LPHNPs-VP was observed at 6 h post IP injection, as shown in Fig. 5b and c. It is worth noting that while the signal intensity gradually reduced over time, it remained detectable until 24 h (Fig. 5b). Taken together, we selected the IP injection method with a 6 h interval to ensure the maximal anti-metastatic RA-PDT effect.

We first examined the therapeutic effect of RA-PDT on the primary tumour growth. As shown in Fig. 5d, the RA-PDT group exhibited a considerably lower bioluminescence signal level from primary tumour compared to other treatment groups (PBS, FA-LPHNPs-VP, and X-ray) within 18 days post-treatment (p < 0.05; Two-way ANOVA with Tukey's multiple comparison post hoc test), indicating the RA-PDT method led to a statistically significant reduction of the primary tumour growth rate. This finding was further supported by the tumour size and volume data as shown in Figs. S19 and S20, showed a considerable reduction in tumour size compared to control group, with a mean difference of 174.46 mm^3^ (95% CI: 124.02 mm^3^, 224.89 mm^3^; p < 0.05; Two-way ANOVA with Tukey's multiple comparison post hoc test). Additionally, we carried out a quantitative analysis of the relative amount of live and necrotic tissue in the tumours (Fig. S21). The results revealed a markedly higher percentage of necrotic cells in RA-PDT group (53.60% (95% CI: 50.24%, 56.96%)) compared to the other groups: about 20% in both the PBS group (95% CI: 17.76%, 22.25%) and the FA-LPHNPs-VP group (95% CI: 17.58%, 23.10%), and 27.88% in X-ray group (95% CI: 26.09%, 29.67%, p < 0.005). All these findings align with the results obtained from orthotopic mouse model and confirm the therapeutic effect of RA-PDT for the treatment of primary colorectal cancer.

To evaluate the therapeutic effect on lymph node metastases, we conducted immunohistochemistry analysis of inguinal lymph node tissues collected at the end point. As shown in Fig. 5e–g, RA-PDT induced a considerable decrease in mitotic activity and tumour cells invasion levels in inguinal lymph node tissues (1.74% (95% CI: 1.46%, 2.02%) Ki-67-positive and 7.14% (95% CI: 4.94%, 9.34%) cytokeratin-positive cells, respectively), compared with PBS, FA-LPHNPs-VP, and X-ray groups. In addition, fewer mice in RA-PDT groups developed metastatic secondary tumour foci in collected inguinal lymph node tissues (2/10; 20%) at the endpoint compared to PBS, FA-LPHNPs-VP, and X-ray groups (40%; 4/10), as shown in Table S2. These results indicated that RA-PDT effectively inhibits the progress of rectal cancer lymph node metastasis.

## Discussion

In this study, we developed lipid-polymer hybrid nanoparticles loaded with VP (FA-LPHNPs-VP) for RA-PDT metastatic cancer treatment, an approach which avoids the need for metal-based scintillators or radiosensitisers. This nanoplatform consists of a PLGA core, lipid shell, and an active photosensitiser, VP. All components of this nanoplatform are currently used in the clinic, which can enable rapid translation of this treatment to the clinic. The encapsulation efficiency of VP achieved in this study was up to 92.31% (95% CI: 91.10%, 93.51%), ensuring sufficient ROS generation from VP under X-ray radiation. The average size was about 70 nm, which is smaller than the gaps between lymphatic capillary cells.^43^ This feature enables the nanoparticles to accumulate in regional lymph node during systemic circulation by passing through the walls of lymphatic capillaries.

Our results showed a 48-fold increase in intracellular ^1^O_2_ generation when cells are treated with RA-PDT delivered by the prepared nanoplatform, compared to the untreated cells (Fig. S6), This effect was considerably higher than in previous studies where 5-fold increase was observed when co-loading VP and gold nanoparticles in a PLGA platform and a 2-fold increase when co-loading VP and perfluorooctyl-bromide in the same platform.^22^^,^^44^ Such substantial production of ^1^O_2_ could be a key factor contributing to the observed cell death in both *in vitro* (Fig. 3a and b) and *in vivo* (Fig. 4c and d) settings.

Increased cellular accumulation (Fig. S4b) and *in vitro* efficacy (Fig. 3b and d) were achieved by adding folic acid onto the surface of our nanoparticles. Compared with other existing targeting regents for rectal cancer, such as the anti-Epithelial cell adhesion molecule,^45^ Interleukin-4 receptor α,^46^ transferrin receptor,^47^ epidermal growth factor,^48^ αvβ5 integrin,^49^ hyaluronic acid,^50^ and chitosan,^51^ folic acid is more cost-effective and folic acid conjugated lipids (such as DSPE-PEG-Folic acid) are already commercially available, avoiding the need for additional conjugation steps and minimising the synthesis complexity.^52^ While our result demonstrated the higher cellular uptake of folic acid-modified nanoparticles (Fig. S4b), it was still slightly lower than previous studies (1.6 vs. 2 times).^53^ This was likely due to a lower amount of folic acid used in our study (5%), while at least 25% of folic acid was conjugated with the nanoparticles reported by other studies.^53^^,^^54^ Nevertheless, the targeting capability of our nanoparticles enhances the specificity of RA-PDT, allowing for a more precise targeting of cancer cells.

To date, the majority of published studies have explored *in vivo* RA-PDT effect using subcutaneous models bearing a primary tumour. In these studies, authors commonly utilised either an intratumour injection^18^^,^^21^^,^^22^^,^^25^ or multiple fractions of X-ray radiation.^17^^,^^19^ It is important to note that these studies did not mimic clinically relevant settings, potentially limiting the translatability of RA-PDT. Additionally, we have not found any prior studies specifically focused on the application of RA-PDT of lymph node tumour treatment. This observation is particularly meaningful as it is estimated that metastasis is the main factor responsible for about 90% of all cancer deaths.^55^

We developed a clinically relevant model where rectal cancer cells were directly introduced in the mouse rectal mucosa. We also used a separate mouse model bearing lymph node metastasis for efficacy assessment of our RA-PDT treatment. The combination of these two models marks our pioneering exploration of RA-PDT for both primary and metastatic tumours. In our study, we used a single-dose treatment (single injection of FA-LPHNPs-VP and a single fraction of radiation) to achieve marked primary tumour inhibition in an orthotopic and lymph node metastatic model (Fig. 4a and b, Fig. 5d, Figs. S19 and S20). Such tumour control is consistent with reduced cell viability (Fig. 3b) and increased cellular apoptosis/necrosis (Fig. 3d) and necrotic cancer cells (Fig. 4d and Fig. S21). Furthermore, we demonstrated impediment of the progression of lymph node metastasis (Table S2). We showed that cell death (tumour necrosis) was increased, and cell proliferation (Ki-67 expression) was decreased in the RA-PDT treated tumours. Importantly, this was achieved with excellent biosafety and biocompatibility, as displayed in Figs. S14 and S22, providing the possibility of treatment plan adjustment (such as repeat injection and radiation exposure) for further enhancing efficacy and addressing possible tumour regrowth.

However, we acknowledge several limitations of this study. Firstly, the treatment was evaluated using cell line-derived models, which exhibit limited immune responses compared to those observed in humans. Therefore, further validation in patient-derived xenograft models is necessary to better mimic the genetic and immunological complexity of human tumours. Secondly, the radiation applied in this study was designed for preclinical research settings, and the therapeutic efficacy may differ when translated to clinical scenarios using a standard linear accelerator. Additionally, due to the involvement of photosensitisers in this formulation, potential adverse effects of RA-PDT may be similar to those associated with conventional PDT. Cost considerations are also a critical aspect of future clinical development. Our work indicates that the manufacturing process of the RA-PDT therapeutics may be cost-effective. The considerations of the clinical scenarios of RA-PDT applicability are outside the scope of our work, but we note that RA-PDT method is designed to integrate seamlessly into existing radiotherapy workflows with minimal disruption. Patients would only require a simple intravenous or intraperitoneal injection of the nanoparticle suspension prior to their routine radiotherapy sessions. This approach enhances patient acceptability by avoiding additional radiation exposure while maintaining therapeutic efficacy.

In summary, the nanoparticle formulation developed in this study is made from clinically approved components using a straightforward synthesis method. When delivered through this nanoplatform, RA-PDT effectively inhibited the primary tumour and efficiently suppressed lymph node tumours *in vivo*. These findings offer a new perspective for radio-dynamic therapy in the direction of clinical translation.

## Contributors

R.S., S.N., T.H., C.P., and T.A.B. performed experiments, analysed the data and revised the manuscript. F.D. assisted with data analysis and revised the manuscript. R.S. and W.D. design the experiments, analysed the data, organised the figures, wrote the original manuscript and revised the manuscript. W.D., A.E., and E.M.G. conceived the experiments and revised the manuscript. E.M.G. and W.D. designed the experiments, funding supported and supervised the overall study. W.D. had final responsibility for the decision to submit for publication. All authors reviewed, critiqued, and provided comments to the text. R.S. and W.D. had full access to all the data in the study and verified the data. All authors read and approved the final version of the manuscript.

## Data sharing statement

The data supporting the conclusions of this study has been included in the paper, with the underlying data for graphs and charts provided in the Supplementary Data. Any additional data supporting the conclusions of this study are available from the corresponding authors Wei Deng (Wei.Deng@uts.edu.au) and Ewa M. Goldys (e.goldys@unsw.edu.au) upon reasonable request.

## Declaration of interests

R.S., A.E., E.M.G., and W.D. have applied a provisional patent for this work. The other authors report no conflicts of interest in this work.

## Appendix ASupplementary data

Supplementary Figs and Tables

Animal Experimentation Approval Information

Reagent Validation File
