# Supplementary material for: Unlocking the in vivo therapeutic potential of radiation-activated photodynamic therapy for locally advanced rectal cancer with lymph node involvement
Source: eBioMedicine. 2025 May 12;116:105724. doi: 10.1016/j.ebiom.2025.105724 (PMC12141937; doi:10.1016/j.ebiom.2025.105724)
Supplement: Supplementary Figs and Tables [file mmc1.docx]

**Unlocking the *in vivo* therapeutic potential of radiation-activated photodynamic therapy for locally advanced rectal cancer with lymph node involvement**

Rui Sang ^1,2^, Sheri Nixdorf ^2^, Tzongtyng Hung ^3^, Carl Power ^3^, Fei Deng ^1^, Thuy Anh Bui ^2^, Alexander Engel ^4,5^, Ewa M. Goldys ^1,*^ and Wei Deng ^2,*^

^1^ Graduate School of Biomedical Engineering, ARC Centre of Excellence in Nanoscale Biophotonics, Faculty of Engineering, UNSW Sydney; Sydney, NSW, 2052, Australia

^2^ School of Biomedical Engineering, University of Technology Sydney; Sydney, NSW, 2007, Australia

^3^ Biological Resources Imaging Laboratory, UNSW Sydney; Sydney, NSW, 2052, Australia

^4^ Sydney Medical School, University of Sydney; Sydney, NSW, 2050, Australia

^5^ Department of Colorectal Surgery, Royal North Shore Hospital, St Leonards; Sydney, NSW, 2065, Australia

*Corresponding author. Email: e.goldys@unsw.edu.au and Wei.Deng@uts.edu.au

**Results**


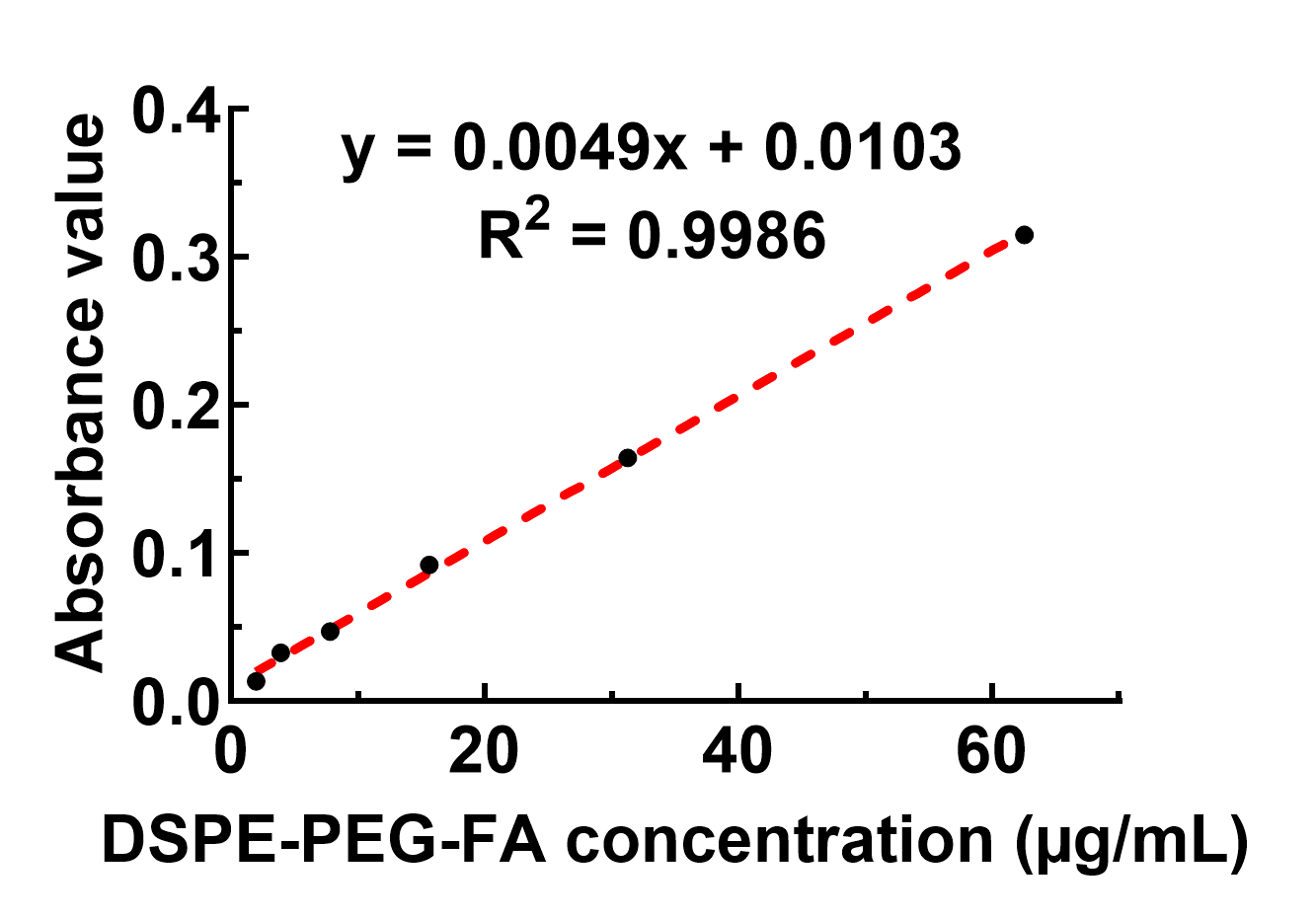


**Fig. S1** Standard curve of DSPE-PEG-FA in the solution of water and acetonitrile with volume ratio 1:1.


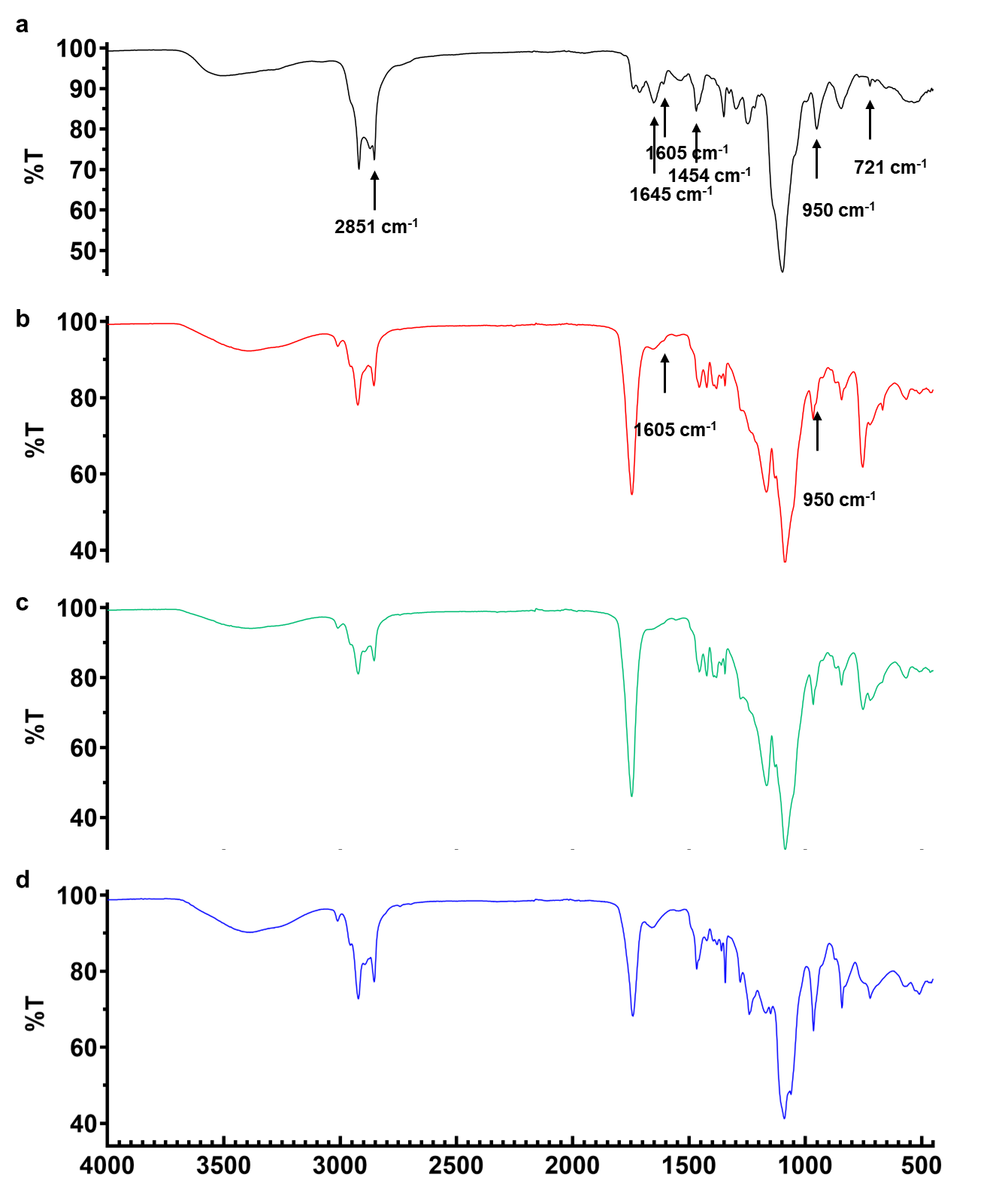


**Fig. S2** FTIR spectra of (a) DSPE-PEG-FA, (b) FA-LPHNPs-VP, (c) LPHNP-VP and (d) LPHNPs.

**
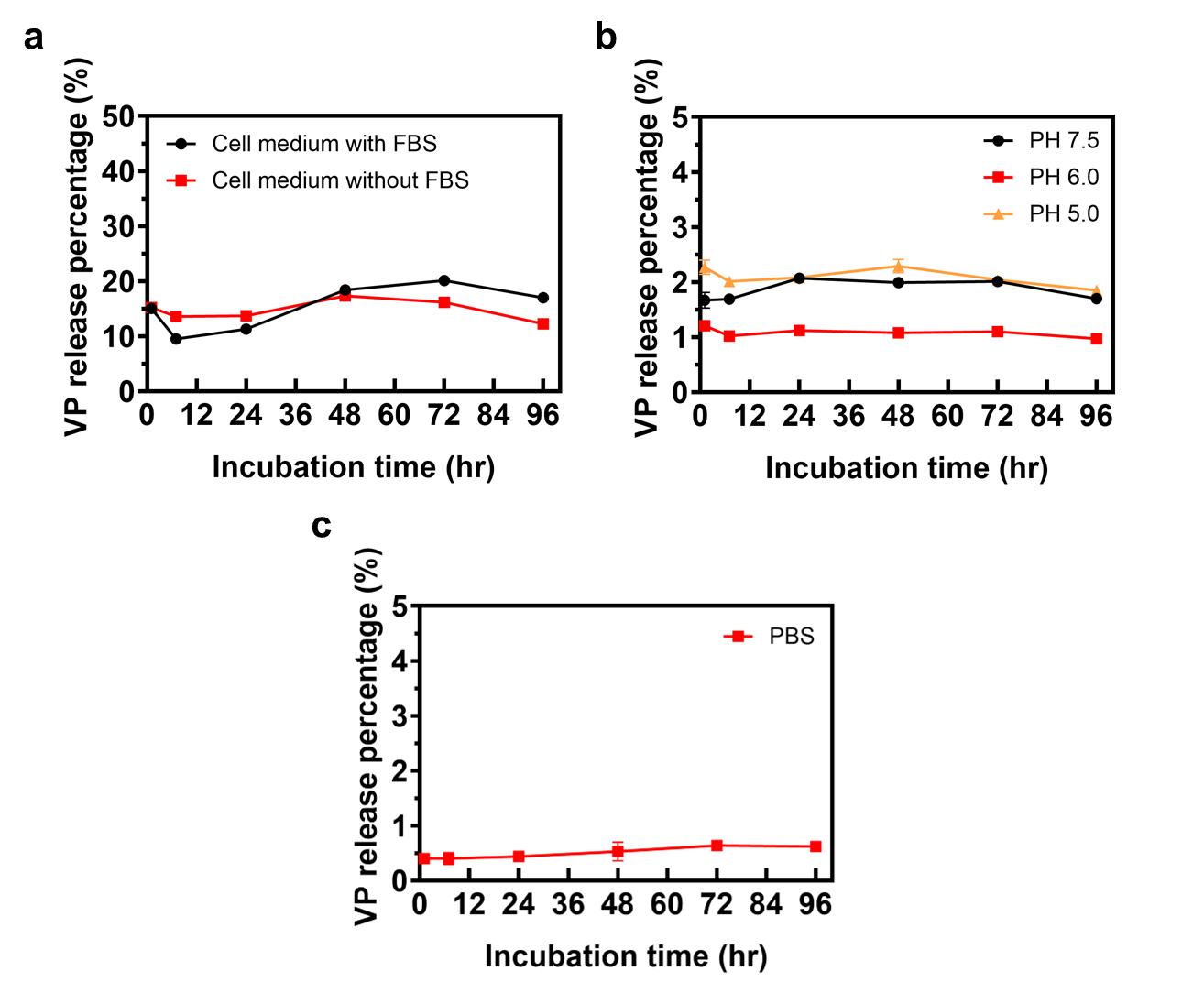
**

**Fig. S3** The stability assessment of FA-LPHNP-VP in (a) cell medium with or without FBS (McCoy’s 5A Medium), (b) different pH buffer and (c) PBS within 96 hrs, Mean ± SD of three independent experiments.

**Cancer cell targeting capacity, ^1^O_2_ and intracellular ROS generation of FA-LPHNPs-VP**

Targeting ability of FA-LPHNPs-VP was evaluated by comparing its cellular uptake activities with the unmodified control (LPHNPs-VP) and normal human colon cells (CCD841). FA-LPHNPs-VP demonstrated enhanced targeting of rectal cancer cells (HCT116) by significantly increasing cellular uptake compared to unmodified nanoparticles (LPHNPs-VP), with up to 61.94% (95% CI:54.81%, 69.06%) higher mean fluorescence intensity at 2 hr after treatment (Fig. S4a-b and Fig. S5a). Comparatively, CCD841, which express low levels of folate receptors, did not show any differential accumulation between these two nanoparticle samples (Fig. S4c-d and Fig. S5b). These results suggest that folic acid modification enhanced active targeting capability of our nanoparticles, resulting in a greater internalisation speed of FA-LPHNPs-VP in HCT116 cells compared with CCD841.

The production of cytotoxic ROS, such as ^1^O_2_, is a key factor in the RA-PDT effect. Therefore we first assessed the ^1^O_2_ production by FA-LPHNPs-VP and LPHNPs-VP in solution. As shown in Fig. S4e, ^1^O_2_ enhancement produced by these two nanoparticles initially increased with X-ray irradiation dose increasing from 0 to 4 Gy but plateaued at 6 Gy. Therefore, 4 Gy was selected for the following intracellular ROS assessment. In addition, FA-LPHNPs-VP exhibited higher ^1^O_2_ generation in comparison to LPHNPs, with approximately 106.32% (95% CI:106.05%, 106.58%) increase in SOSG fluorescence intensity following 4 Gy exposure. We then evaluated *in vitro* ROS generation by using ROS assay kits and comparing different groups: X-ray alone to assess radiation-induced ROS; LPHNPs alone to determine baseline nanoparticle effects; LPHNPs & X-ray to evaluate nanoparticle-radiation interactions; LPHNP-VP alone to examine VP’s role in ROS production; LPHNP-VP & X-ray to assess RA-PDT synergy; FA-LPHNPs-VP alone for tumour-targeting efficiency; and FA-LPHNPs-VP & X-ray to test the optimised RA-PDT strategy.

As shown in Fig. S4f and g, a weak and comparable DCFDA fluorescence signal was observed for control group and the group treated with LPHNPs alone, suggesting that LPHNPs did not contribute to intracellular ROS generation. Additionally, a slight increase in fluorescence was detected for 4 Gy X-ray alone, and for LPHNPs & 4 Gy treated cell groups, indicating that 4 Gy X-ray has a minor impact on ROS production in HCT116 cells. The cell groups treated with LPHNPs-VP and FA-LPHNPs-VP had comparable fluorescence signal levels, suggesting a similar amount of ROS produced. However, the fluorescence intensity from cells treated with LPHNPs-VP and FA-LPHNPs-VP in combination with 4 Gy X-ray showed statistically significant increases compared to that of the cell alone group (p < 0.005; Two-way ANOVA with Tukey’s multiple comparison post hoc test), increasing by factors of 5.46 (95% CI:5.24, 5.68) and 8.64 (95% CI:7.99, 9.29), respectively. This indicates that a substantial amount of ROS was generated through RA-PDT. SOSG fluorescence signal further confirmed substantial intracellular ^1^O_2_ generation in FA-LPHNPs-VP & 4 Gy treated cell groups, highlighting the potent RA-PDT efficacy of FA-LPHNPs-VP (Fig. S6). These results indicated that FA-LPHNPs-VP efficiently generated intracellular ROS and ^1^O_2_ in combination with X-ray irradiation, providing conditions which may trigger cancer cells death.


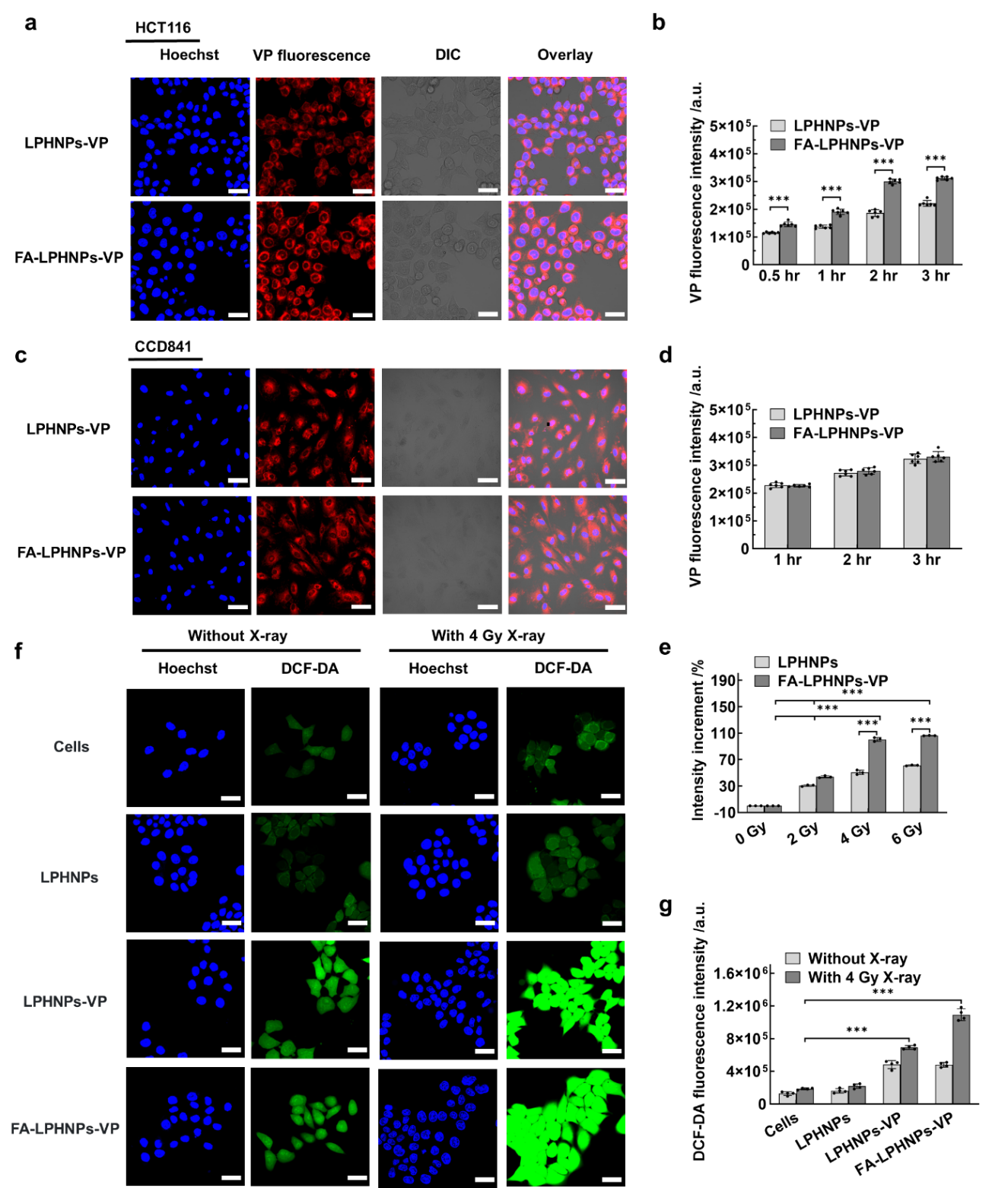


**Fig. S4** **Cellular uptake activity and ROS generation assessment.** Representative confocal microscope images of (a) HCT116 and (c) CCD841 cells incubated with LPHNPs-VP and FA-LPHNPs-VP for 2 h. The scale bar is 30 μm. Quantitative analysis of VP fluorescence from LPHNPs-VP and FA-LPHNPs-VP in (b) HCT116 and (d) CCD841 (Two-way ANOVA with Tukey’s multiple comparison post hoc test); (e) The intensity increments of SOSG fluorescence assessed in LPHNPs-VP and FA-LPHNPS-VP aqueous solution under different X-ray irradiation dose (Two-way ANOVA with Tukey’s multiple comparison post hoc test); (f) Intracellular ROS production of cell groups treated with various conditions. Scale bar is 30 μm. G. ROS generation analysis as indicated in Fig. S4f (Two-way ANOVA with Tukey’s multiple comparison post hoc test). The calculation of every group included more than 60 cells. *p ＜0.05, **p ＜0.01 and ***p ＜0.005; Mean ± SD of three independent experiments where each dot represents the mean of 2-3 replicates.

**
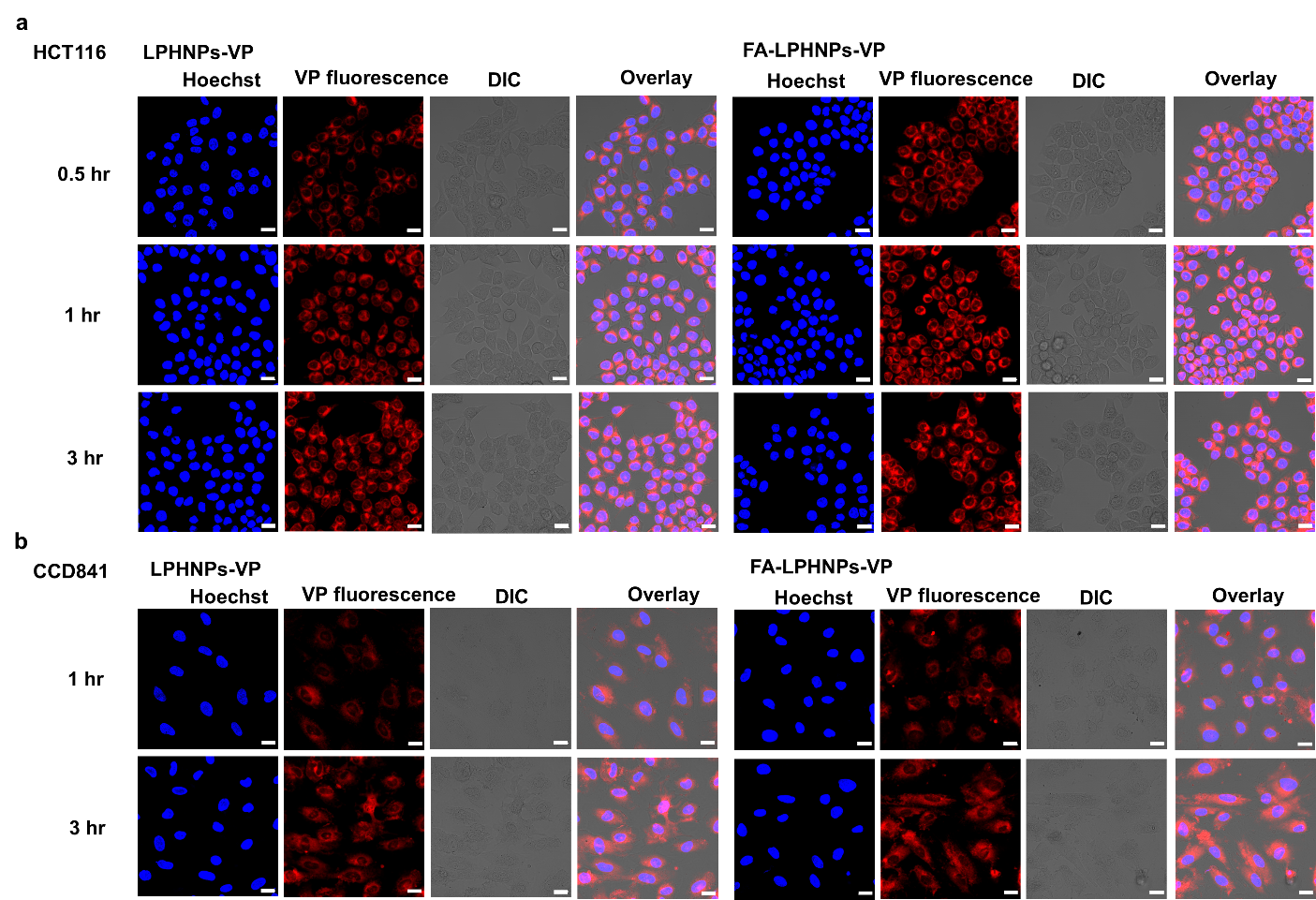
**

**Fig. S5** Representative confocal microscope images of (a) HCT116 cells and (b) CCD841 cells after incubation with LPHNPs-VP and FA-LPHNPs-VP at different time points. The scale bar is 30 µm.


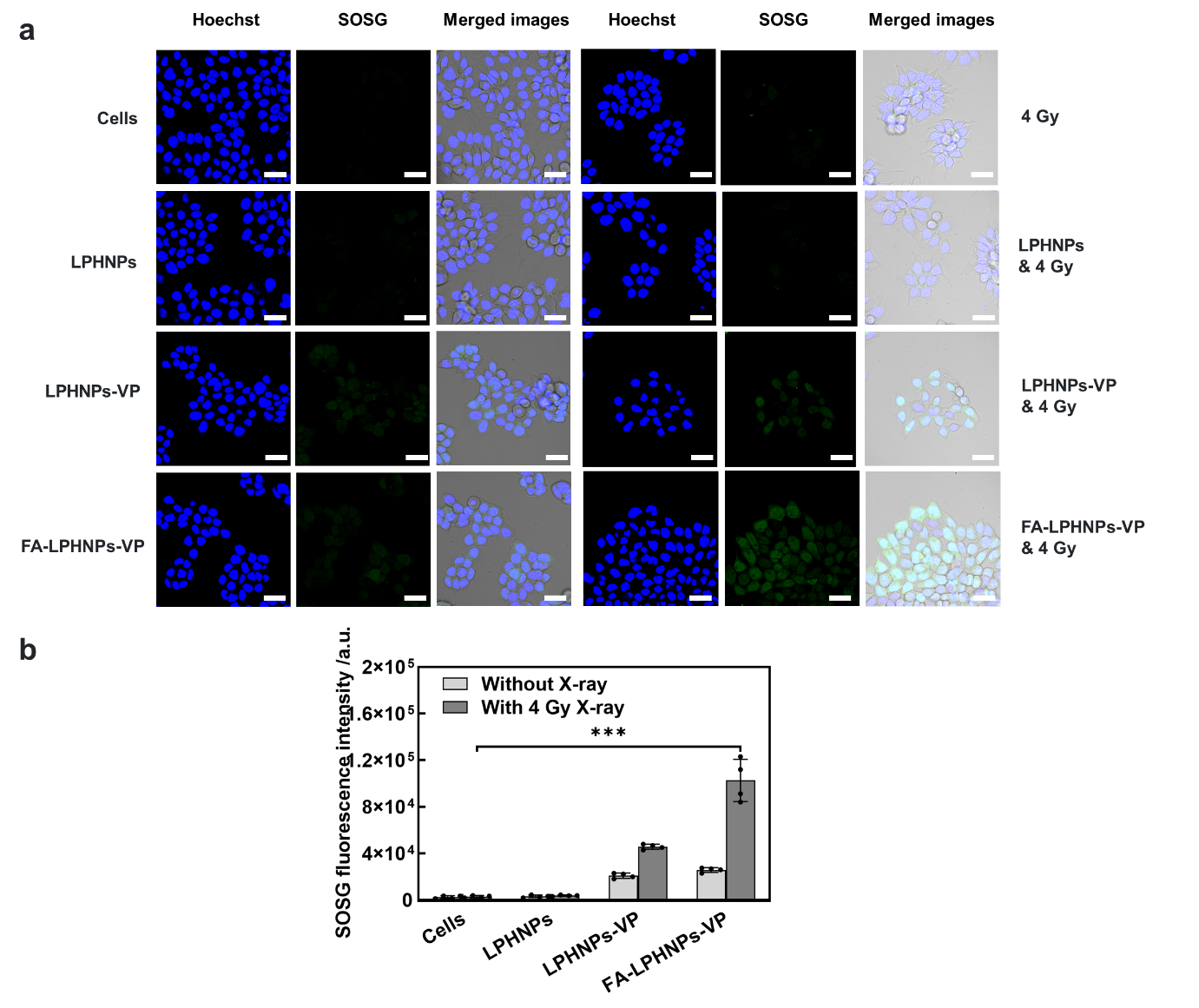


**Fig. S6** (a) Intracellular ^1^O_2_ generation form nanoparticles with and without X-ray irradiation. Scale bar is 30 μm; (b) Quantitative analysis of ^1^O_2_ generation from each group (Two-way ANOVA with Tukey’s multiple comparison post hoc test, Mean ± SD of three independent experiments where each dot represents the mean of 2-3 replicates, ***p ＜0.005).

*In vitro* dark cytotoxicity of FA-LPHNPs-VP was examined by using the MTS assay. As shown in Fig. S7, there was no statistically significant effect on cell viability at concentrations of FA-LPHNPs-VP up to 514.80 μg/mL compared with the control group (p > 0.05; Two-way ANOVA with Tukey’s multiple comparison post hoc test), suggesting good biocompatibility of these nanoparticles. Therefore, the following *in vitro* experiments were conducted with 514.80 μg/mL of FA-LPHNPs-VP.


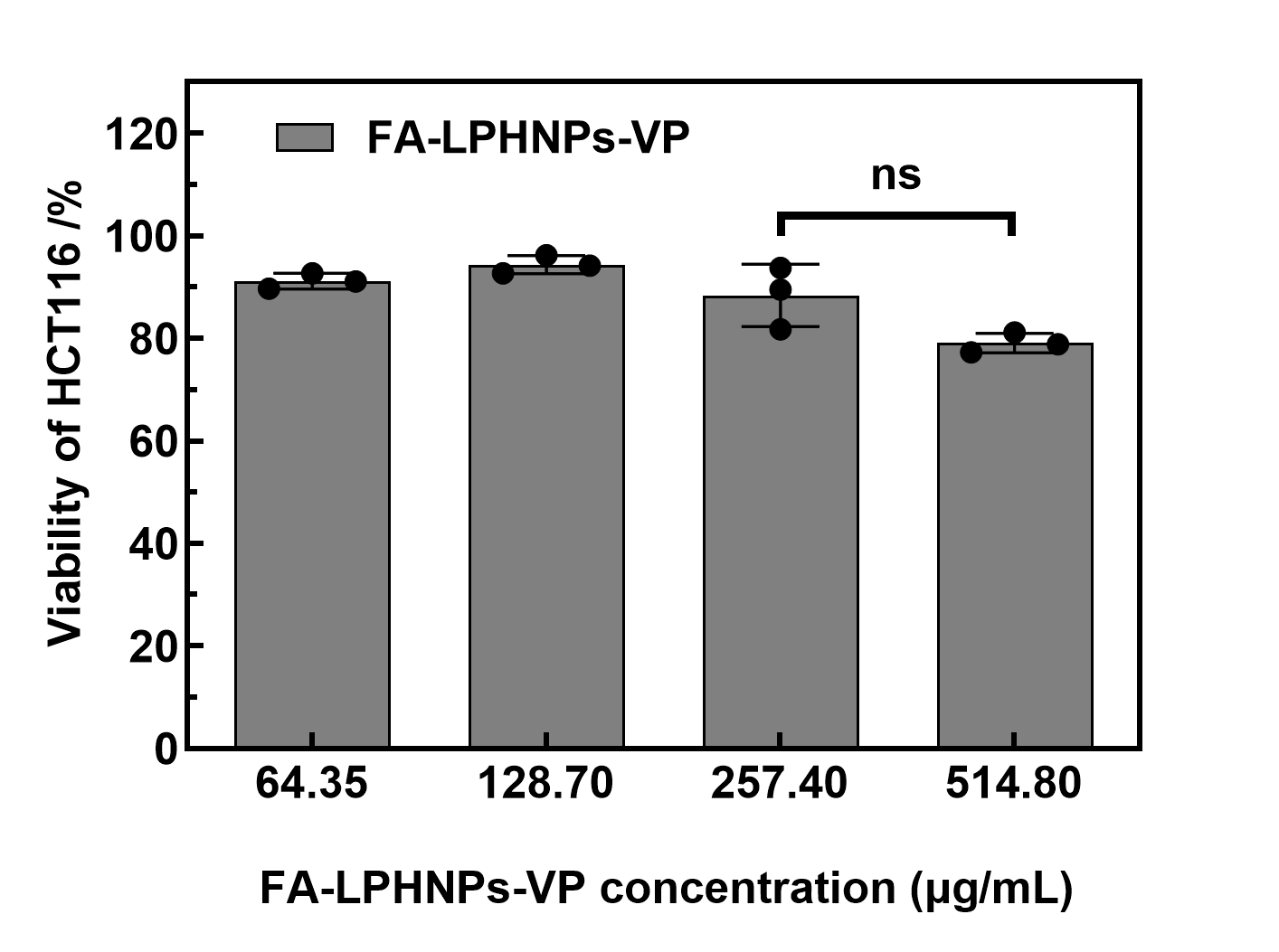


**Fig. S7** The viability percentage of HCT116 at 24 hr after incubation with FA-LPHNPs-VP at different concentrations. (Two-way ANOVA with Tukey’s multiple comparison post hoc test, Mean ± SD of three independent experiments, ns: p > 0.05).


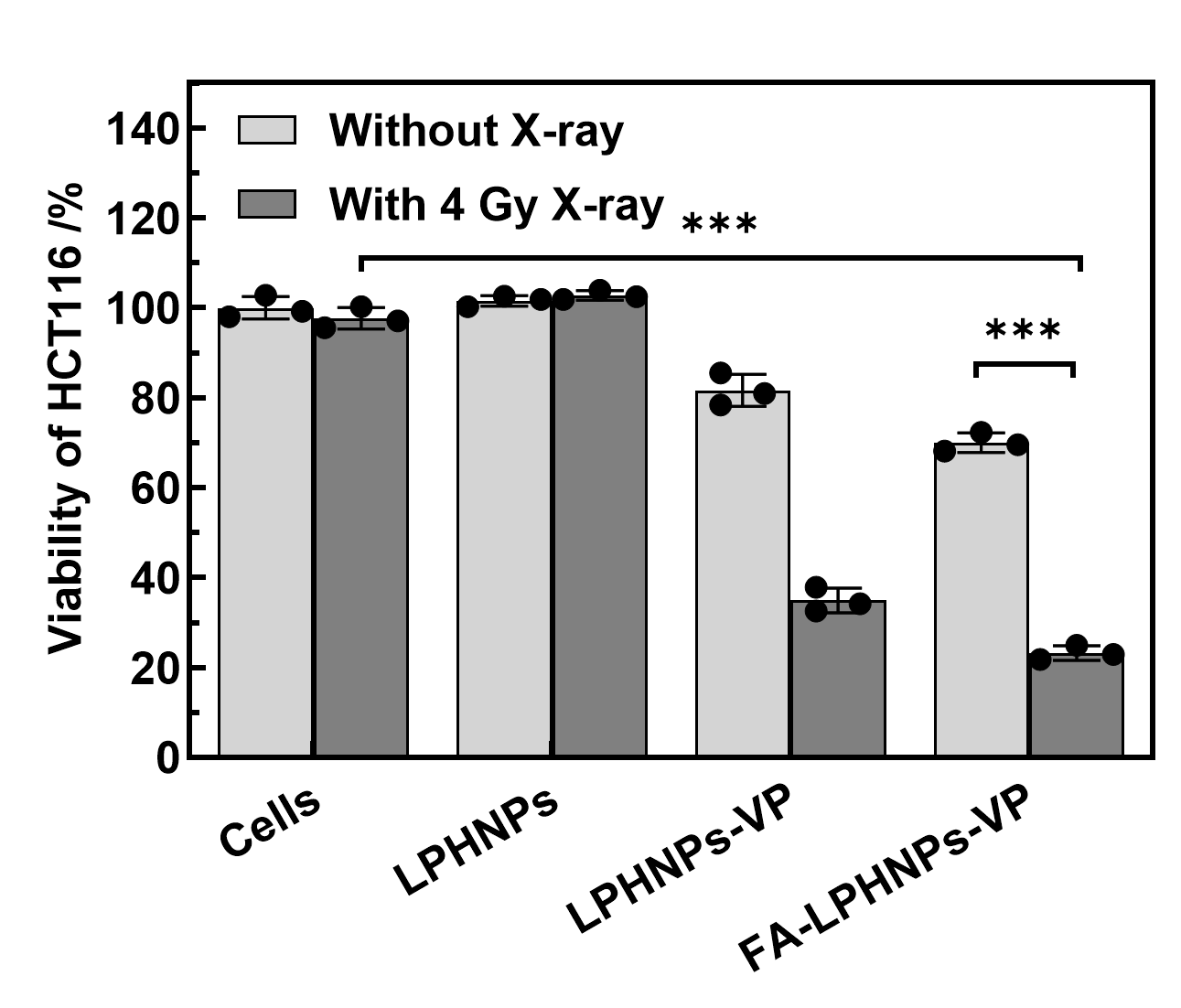


**Fig. S8** The viability percentage of HCT116 cells at 24 hr after different treatments as indicated via MTS assay. (Two-way ANOVA with Tukey’s multiple comparison post hoc test, Mean ± SD of three independent experiments, ***p ＜0.005).


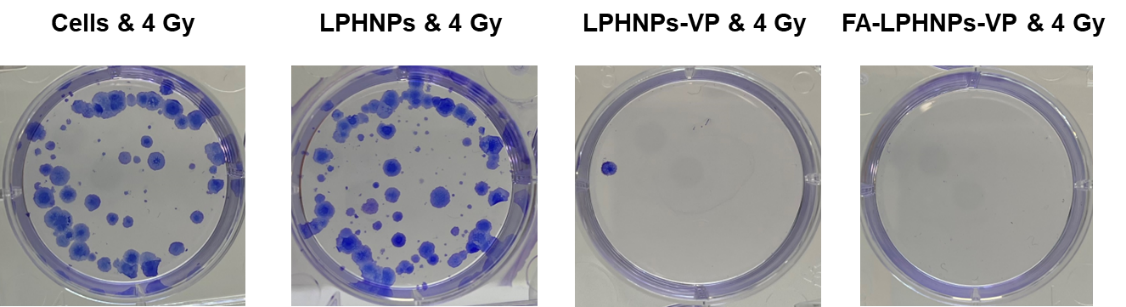


**Fig. S9** Representative photographs of clonogenic survival assay of stained colonies at 14 days after 4 Gy X-ray irradiation with or without nanoparticles treatment (n=three independent experiments).


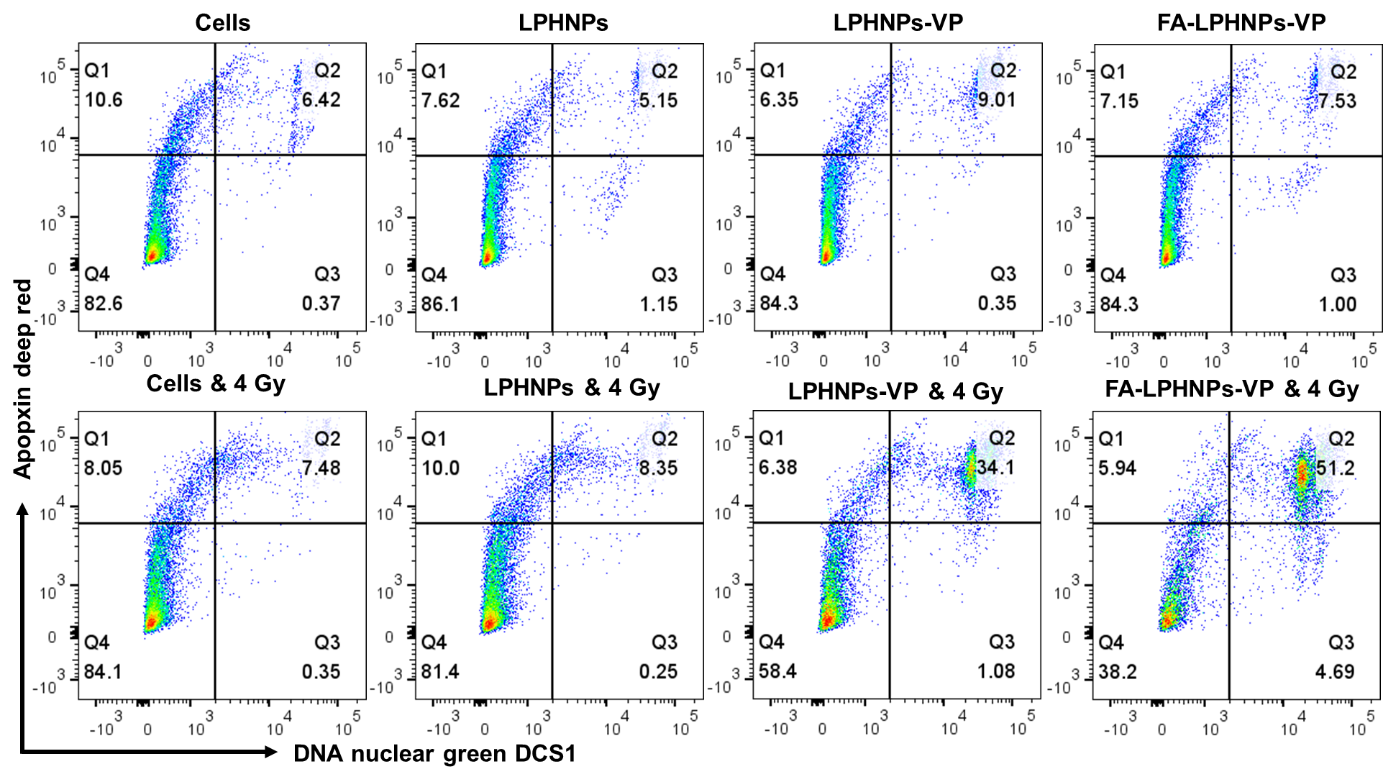


**Fig. S10** Representative FACS plots gated on DNA nuclear green DCS1-positive (necrotic) cells and Apopxin deep red-positive (apoptotic) cells at 24 hr after different treatments (n=three independent experiments).


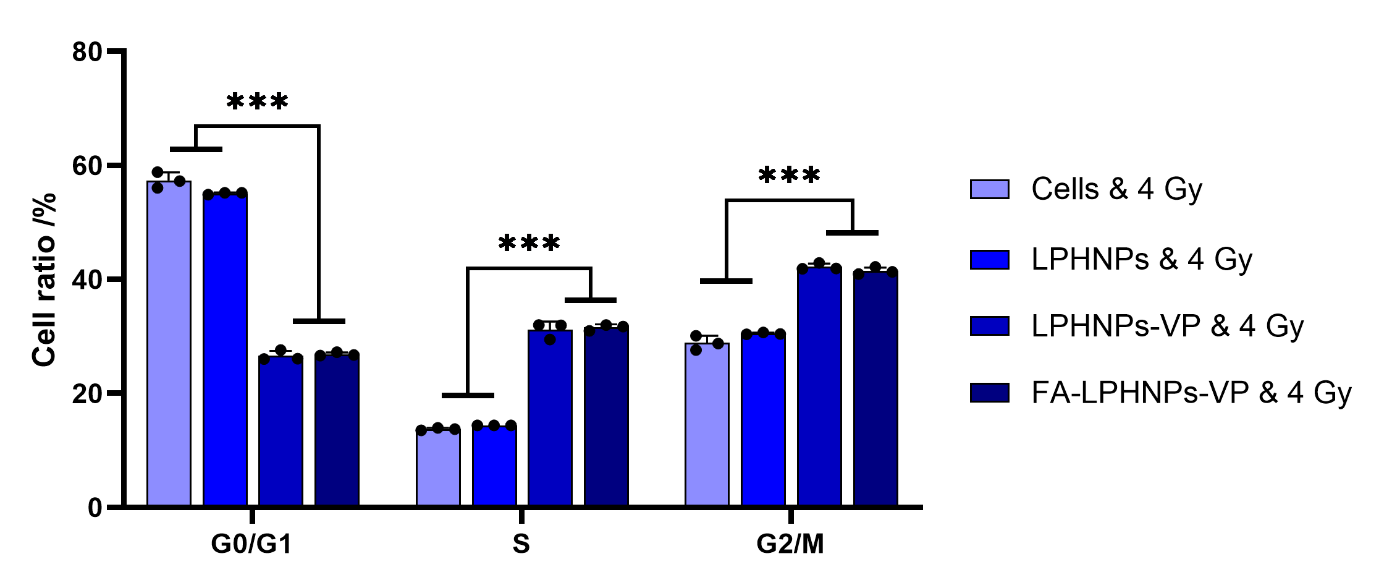


**Fig. S11** The proportion of cell population in G0/G1, S and G2/M phases at 48 hr for X-ray irradiated groups (Two-way ANOVA with Tukey’s multiple comparison post hoc test, Mean ± SD of three independent experiments).

**Assessment of nanoparticles’ biodistribution and safety profile in an orthotopic mouse model**

To evaluate the RA-PDT efficacy against rectal tumour growth, an orthotopic rectal tumour model was established using HCT-116-Luc2 cells, with tumour growth monitored via bioluminescence and ultrasound imaging (Fig. S12a-b and Fig. S13). Tumour volume measured with the ultrasound correlated well (R^2^=0.9090) with the bioluminescence signal (Fig. S12c), enabling us to analyse tumour size and determine suitable time points for the treatments. The FA-LPHNPs-VP nanoparticle biodistribution analysis revealed that maximal tumour accumulation of FA-LPHNPs-VP was observed at 4 hr post intravenous (IV) injection, and they were cleared from the mouse body at 24 hr (Fig. S12d-e). Therefore, the 4-hr time point post IV injection of FA-LPHNPs-VP was selected for X-ray delivery in RA-PDT treatment. In addition, the VP concentration in frozen tumour tissues at 4 hr were measured after extraction VP into acetonitrile, revealing that the VP concentration in tumour tissues at 4 hours post-IV injection was 462.54 ng per gram of tumour tissue (95% CI: 419.27 ng/g, 505.81 ng/g), corresponding to approximately 0.3% of the injected dose.

To gain further insight into the biosafety profile of nanoparticles, we conducted the biosafety measurement on these nanoparticles. No behavioural changes or statistically significant weight loss were observed in any of the mouse groups during the 72-hour or 14-day period following the single (Fig. S14a) or multiple injections (Fig. S14b) (p > 0.05; Two-way ANOVA with Tukey’s multiple comparison post hoc test). Additionally, the H&E staining of major organs including heart, lungs, liver, spleen and kidneys showed no obvious histological changes within a 7-day period after four repeated FA-LPHNPs-VP injections, indicated that FA-LPHNPs-VP did not cause obvious systemic toxicity (Fig. S14c). Furthermore, haematological analysis of whole blood following four repeated administrations at three administrative doses (12.5, 25 and 50 mg/kg) of FA-LPHNPs-VP showed no statistically significant effect on blood composition (p ˃ 0.05; Two-way ANOVA with Tukey’s multiple comparison post hoc test), even at the highest tested dose (50 mg/kg) (Fig. S14d-f and Table S1). In addition, no statistically significant difference was observed in secretion of three major inflammatory cytokines, namely TNF alpha, INF gamma or IL-6, in serum at various time points (30 min, 2 hr, 5 hr, 24 hr and 7 days post-injection) (p ˃ 0.05; Two-way ANOVA with Tukey’s multiple comparison post hoc test), indicating that FA-LPHNPs-VP did not notably stimulate an immune response. Overall, these results suggest that FA-LPHNPs-VP had excellent biosafety and biocompatibility confirmed by this study. Based on these *in vivo* toxicity results, a safe dose of FA-LPHNPs-VP equal to 50 mg/kg was chosen for the RA-PDT antitumour experiment.


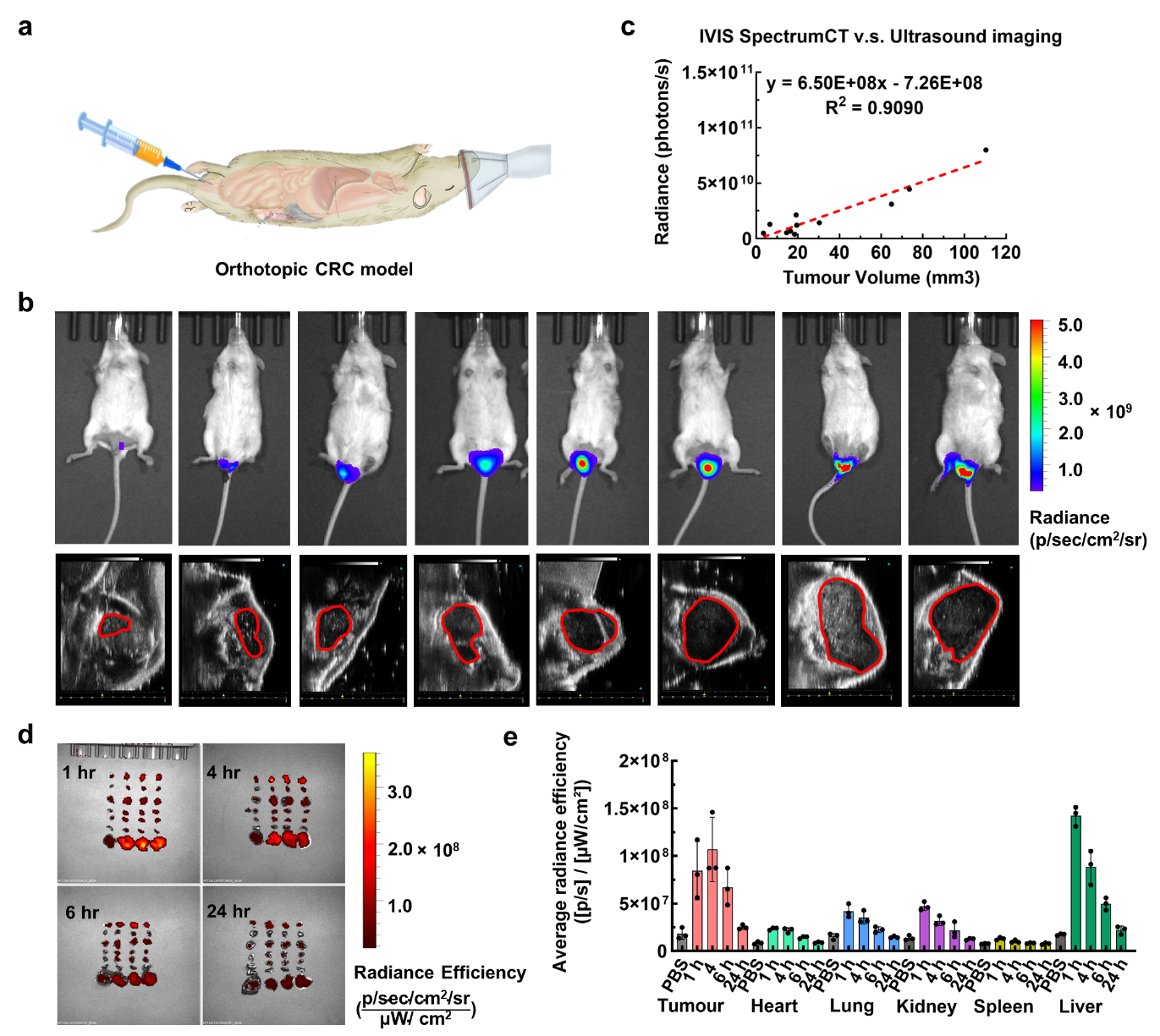


**Fig. S12** ***In vivo* biodistribution of FA-LPHNPs-VP nanoparticles.** (a) Depiction of the intrarectal injection method employed to establish an orthotopic model; (b) Detection of tumour growth using (upper panel) bioluminescence imaging and (lower panel) ultrasound imaging; (c) Correlation between bioluminescence signal of tumours and their volume over time after removing two data point based on the principle of beyond ±3 standard deviations from the mean, R^2^ = 0.9090, n=11; (d) *Ex vivo* optical images of various tissues (from top to bottom: tumour, heart, lung, kidney, spleen, liver) for fluorescent nanoparticles; (e) The quantitative data analysis on biodistribution represented graphically (Two-way ANOVA with Tukey’s multiple comparison post hoc test, n=3 mice per group, Mean ± SD).


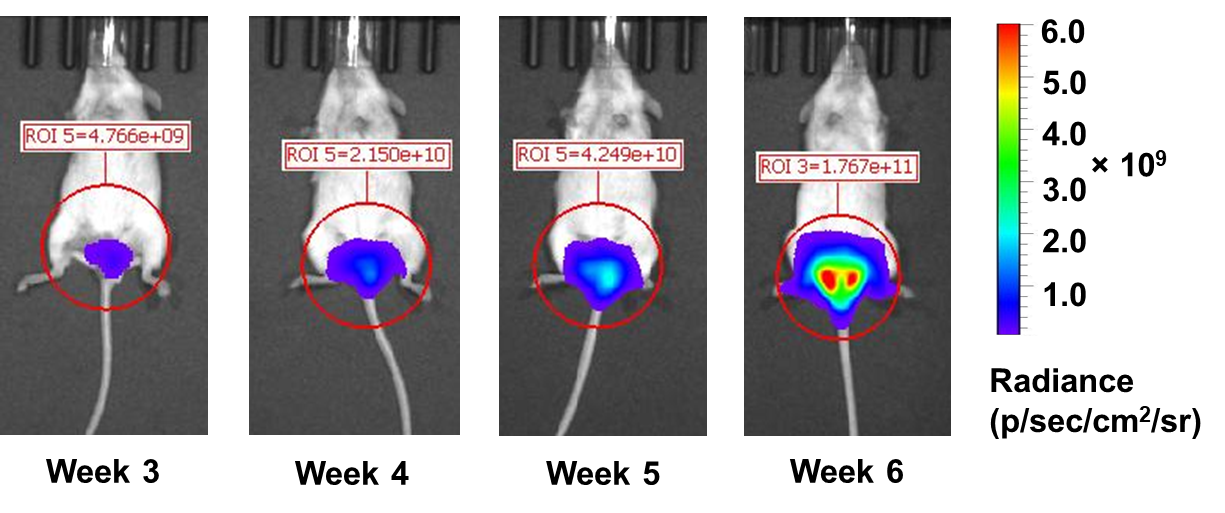


**Fig. S13** Detection of tumour growth using IVIS SpectrumCT imaging between week 3 and week 6.


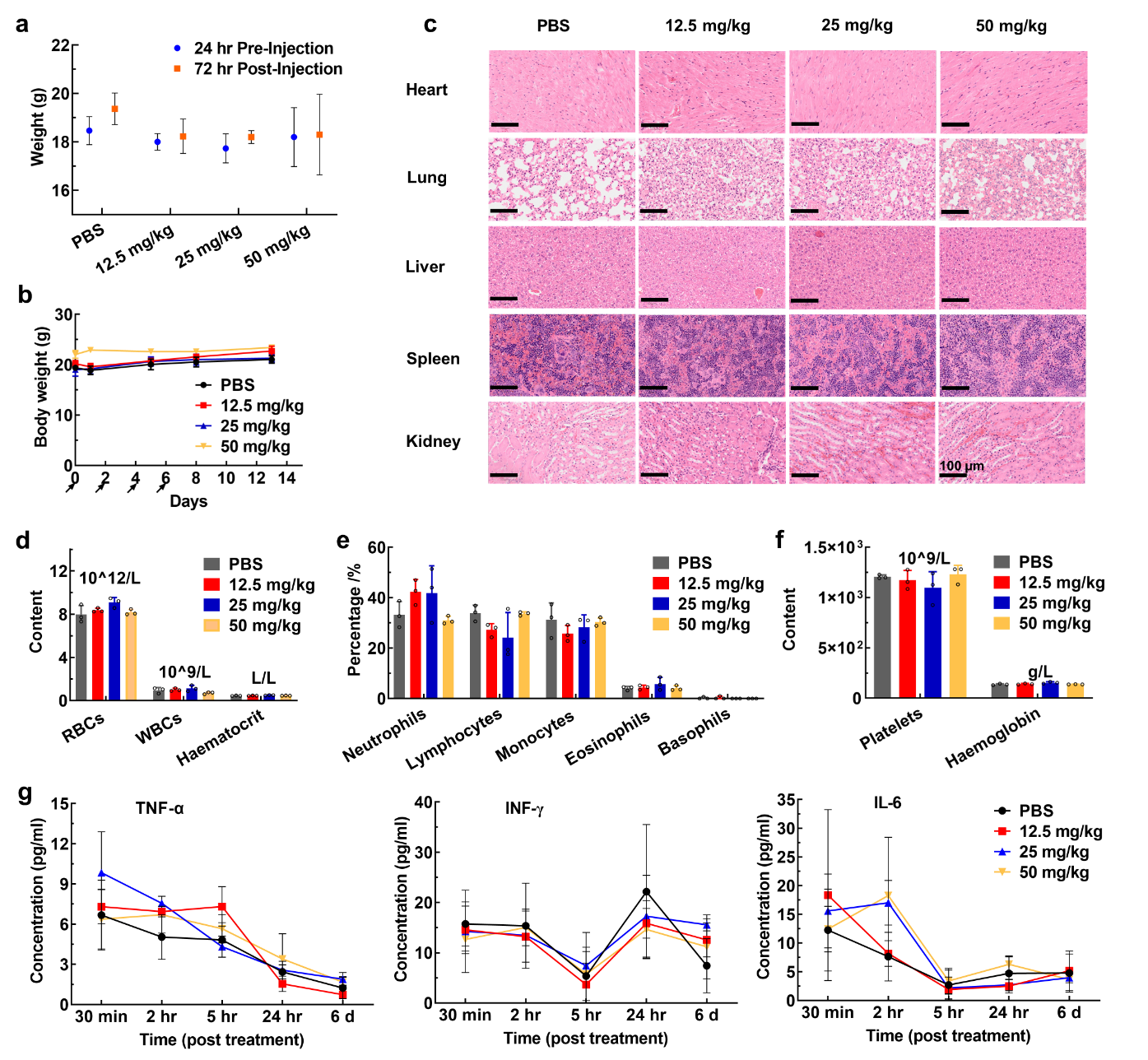


**Fig. S14** **Assessment of *in vivo* FA-LPHNPs-VP’s biosafety.** (a) Weight profile of mice 24 hr before and 72 hr after a single nanoparticle injection (at 12.5, 25 and 50 mg/kg); (b) Body weight profile of mice following multiple FA-LPHNPs-VP IV injections, the arrows indicate injection days; (c) Representative H&E-stained sections of major organs (heart, lung, liver, spleen and kidney) on day 6 after repeat IV FA-LPHNPs-VP injections at increasing concentrations compared to the control group injected with PBS alone. Scale bars, 100 μm; (d-f) Analysis of a range of haematological parameters 6 weeks post-FA-LPHNPs-VP administration (0, 12.5, 25 and 50 mg/kg), p > 0.05; (g) Serum TNF-α, INF-γ and IL-6 cytokine levels at various time points after four separate FA-LPHNPs-VP injections of different doses (0, 12.5, 25 and 50 mg/kg). p > 0.05 (Two-way ANOVA with Tukey’s multiple comparison post hoc test, n=3 mice per group, Mean ± SD).

**Table S1** **The changes in haematological parameters at 6 weeks post the fourth IV administration of FA-LPHNPs-VP at different concentrations (0, 12.5, 25 and 50 mg/kg). The values are expressed as mean (95% CI) (n=3 mice per group).**

| **Parameters** | **PBS control** | **FA-LPHNPs-VP (12.5 mg/kg)** | **FA-LPHNPs-VP (25 mg/kg)** | **FA-LPHNPs-VP (50 mg/kg)** |
| --- | --- | --- | --- | --- |
| **WBCs (10^9/L)** | 0.99 (0.57,1.42) | 1.00 (0.85,1.15) | 1.13 (0.81,1.44) | 0.68 (0.54,0.81) |
| **RBCs (10^12/L)** | 8.06 (7.07,9.05) | 8.36 (8.14,8.58) | 9.08 (8.56,9.61) | 8.26 (7.92,8.61) |
| **Haemoglobin (g/L)** | 135.40 (125.01,145.79) | 140.33 (133.42,147.25) | 153.33 (142.58,164.09) | 138.00 (136.40,139.60) |
| **Haematocrit (L/L)** | 0.44 (0.40,0.47) | 0.45 (0.43,0.47) | 0.49 (0.46,0.52) | 0.45 (0.44,0.46) |
| **Mean Corpuscular Volume (fL)** | 54.62 (51.46,57.78) | 53.87 (52.30,55.43) | 53.60 (53.00,54.20) | 54.50 (52.90,56.10) |
| **Mean Corpuscular Haemoglobin (pg)** | 16.84 (15.96,17.72) | 16.77 (16.30,17.24) | 16.87 (16.52,17.21) | 16.70 (16.22,17.18) |
| **Mean Corpuscular Haemoglobin Concentration (g/L)** | 309.00 (304.07,313.93) | 311.67 (309.94,313.40) | 314.67 (311.21,318.12) | 306.00 (306.00,306.00) |
| **Platelets (10^9/L)** | 1198.00 (1175.35,1220.65) | 1172.67 (1062.81,1282.52) | 1098.00 (917.52,1278.48) | 1206.50 (1085.68,1327.32) |
| **Cell Distribution Width - SD (fL)** | 31.12 (27.93,34.31) | 30.30 (28.31,32.29) | 30.17 (29.32,31.02) | 33.20 (27.12,39.28) |
| **Cell Distribution Width - CV %** | 17.16 (16.94,17.38) | 17.13 (16.62,17.64) | 17.50 (17.11,17.89) | 17.95 (16.27,19.63) |
| **Platelet Distribution Width (fL)** | 7.58 (7.35,7.81) | 7.90 (7.79,8.01) | 8.47 (8.04,8.90) | 8.25 (8.17,8.33) |
| **Mean Platelet Volume (fL)** | 7.58 (7.41,7.75) | 7.70 (7.59,7.81) | 8.00 (7.89,8.11) | 8.05 (7.97,8.13) |
| **Platelet - Large Cell Ratio %** | 7.58 (6.84,8.32) | 8.47 (7.84,9.09) | 10.37 (9.81,10.92) | 10.75 (10.35,11.15) |
| **Procalcitonin %** | 1.31 (1.25,1.37) | 1.19 (1.09,1.30) | 1.12 (0.88,1.36) | 1.26 (1.16,1.36) |
| **Nucleated Red Blood Cell # (10^9/L)** | 0.01 (-0.003,0.027) | 0.02 (0.010,0.023) | 0.02 (0.008,0.031) | 0.02 (0.004,0.036) |
| **Nucleated Red Blood Cell %** | 0.96 (-0.12,2.04) | 1.63 (0.95,2.32) | 1.96 (0.43,3.50) | 3.2 (0.16,6.24) |
| **Neutrophil # (10^9/L)** | 0.32 (0.16,0.47) | 0.43 (0.31,0.55) | 0.49 (0.22,0.75) | 0.22 (0.16,0.27) |
| **Lymphocyte # (10^9/L)** | 0.33 (0.18,0.48) | 0.27 (0.26,0.28) | 0.26 (0.20,0.31) | 0.22 (0.18,0.26) |
| **Monocyte # (10^9/L)** | 0.30 (0.16,0.43) | 0.26 (0.22,0.30) | 0.31 (0.27,0.35) | 0.20 (0.18,0.23) |
| **Eosinophil # (10^9/L)** | 0.04 (0.01,0.07) | 0.04 (0.04,0.05) | 0.07 (0.02,0.12) | 0.03 (0.01,0.05) |
| **Basophil # (10^9/L)** | 0.004 (-0.002,0.010) | 0.003 (-0.003,0.009) | 0.000 (0.000,0.000) | 0.000 (0.000,0.000) |
| **Neutrophils %** | 32.04 (22.73,41.35) | 42.30 (36.68,47.92) | 41.80 (29.50,54.10) | 31.70 (29.78,33.62) |
| **Lymphocytes %** | 33.28 (28.35,38.21) | 27.20 (24.29,30.11) | 24.17 (12.92,35.41) | 33.40 (32.60,34.20) |
| **Monocytes %** | 30.46 (22.73,38.19) | 25.77 (22.16,29.37) | 28.23 (22.61,33.85) | 30.55 (27.91,33.19) |
| **Eosinophils %** | 3.90 (2.06,5.74) | 4.40 (3.43,5.37) | 5.80 (2.90,8.70) | 4.35 (2.83,5.87) |
| **Basophils %** | 0.32 (-0.18,0.82) | 0.33 (-0.32,0.97) | 0.00 (0.00,0.00) | 0.00 (0.00,0.00) |


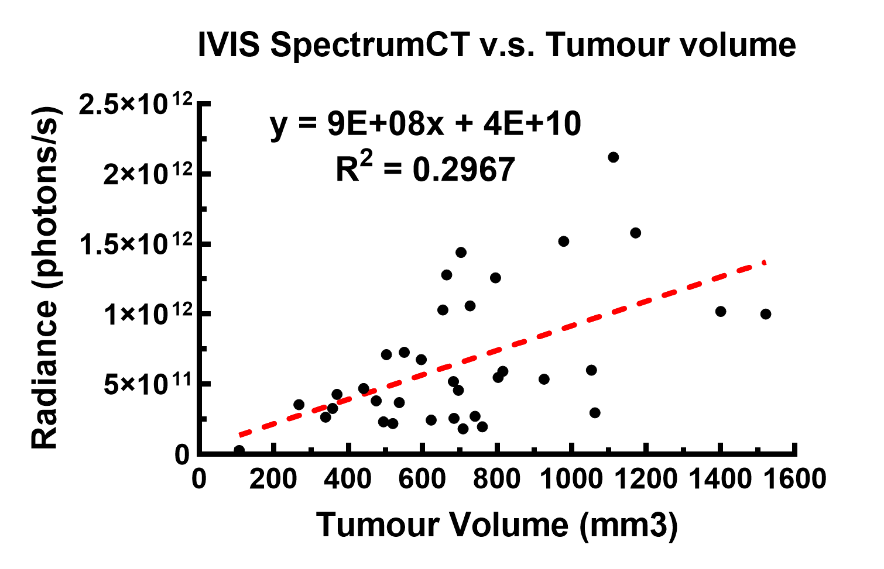


**Fig. S15.** Correlation between bioluminescence signal of tumours at endpoint and their tumour volume when tumour volume is larger than 110 mm^3^, R^2^ = 0.2967, n=35.


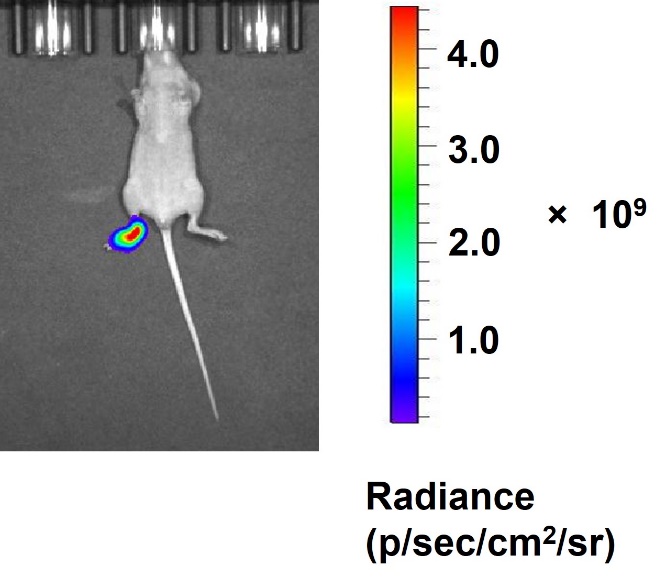


**Fig. S16** *In vivo* bioluminescence signal observed at the footpad on day 21 post-injection of HCT116-Luc2 cells.


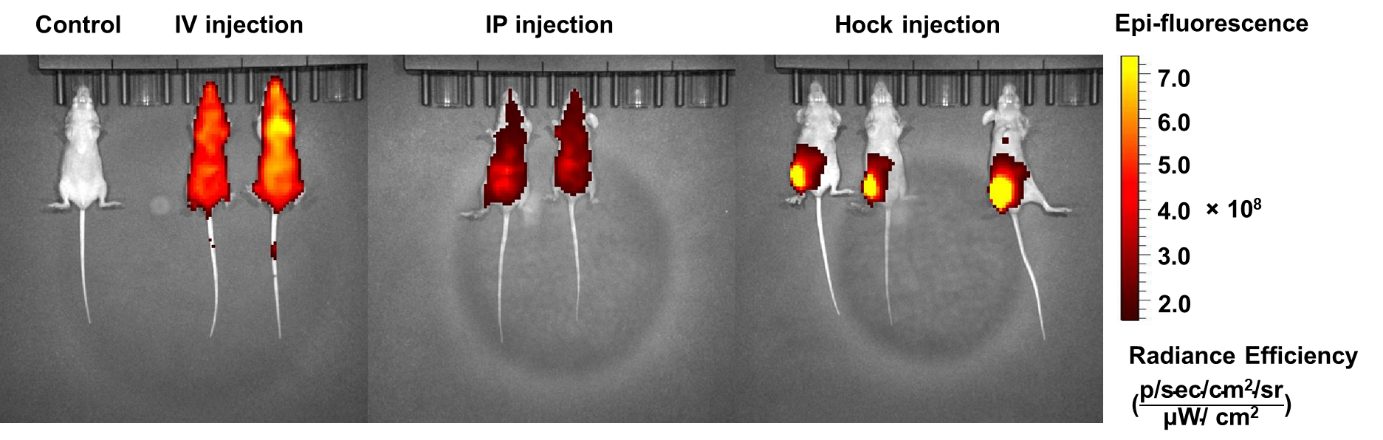


**Fig. S17** *In vivo* fluorescent images conducted by IVIS SpectrumCT at 4 hr post FA-LPHNPs-VP IV, IP and hock injections in HCT116-luc2 lymph node metastasis footpad mouse model.

As shown in Fig. S17, *in vivo* fluorescence imaging revealed that IV and IP injections resulted in a widespread distribution of FA-LPHNPs-VP throughout the body at 4 hrs after FA-LPHNPs-VP injections. In contrast, fluorescence in the hock injection group was primarily localised around the injection site. However, it was challenging to clearly observe nanoparticle accumulation in lymph node tissues at the *in vivo* level due to high background signals from other regions of the body.


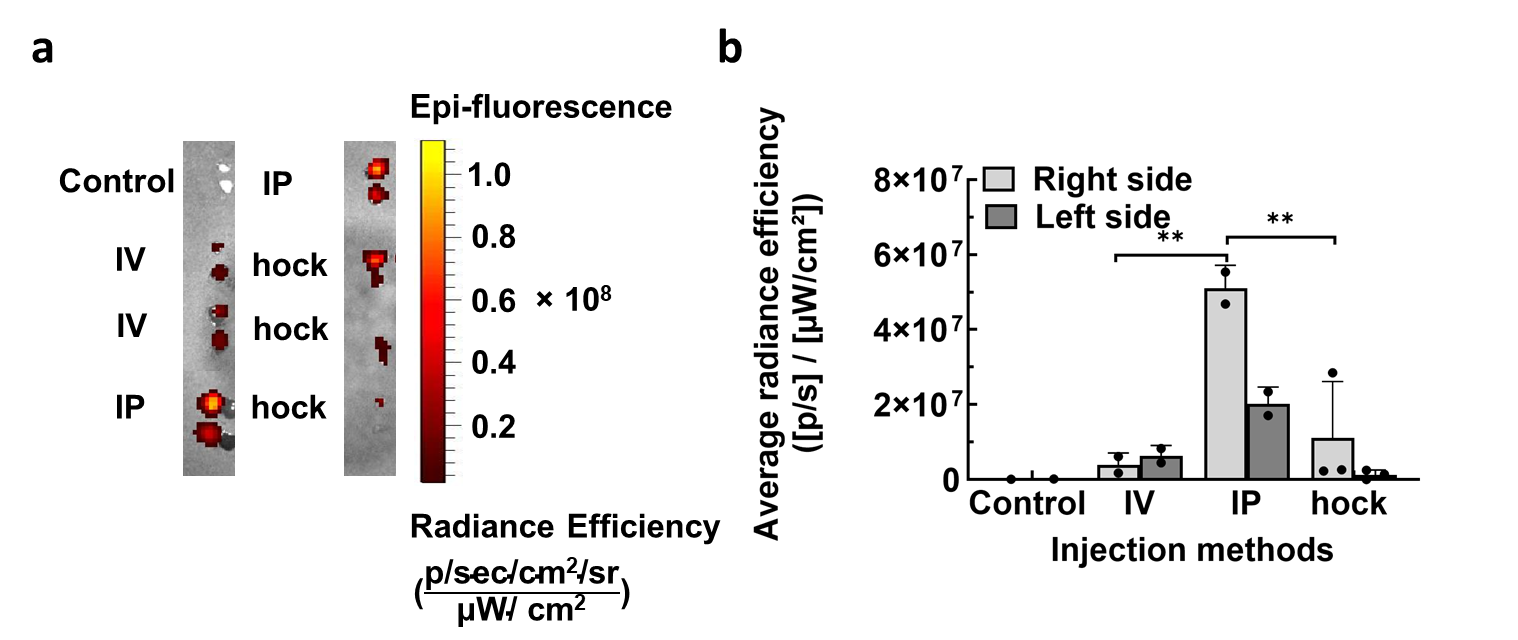


**Fig. S18** (a) *Ex vivo* VP fluorescence signal of the nanoparticles accumulated in inguinal lymph node tissues using different injection methods; (b) The quantitative data analysis of fluorescence signal from lymph node tissues. Right side corresponds to the same side of a primary tumour, while left side refers to the lymph node on the opposite side of a primary tumour (Two-way ANOVA with Tukey’s multiple comparison post hoc test, n=2-3 mice per group, Mean ± SD).


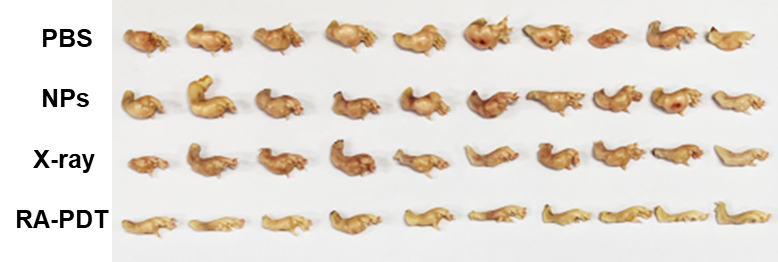


**Fig. S19** Photographs of footpad tissue bearing tumour at the endpoint (Day 28 post-injection of HCT116-Luc2 cells).


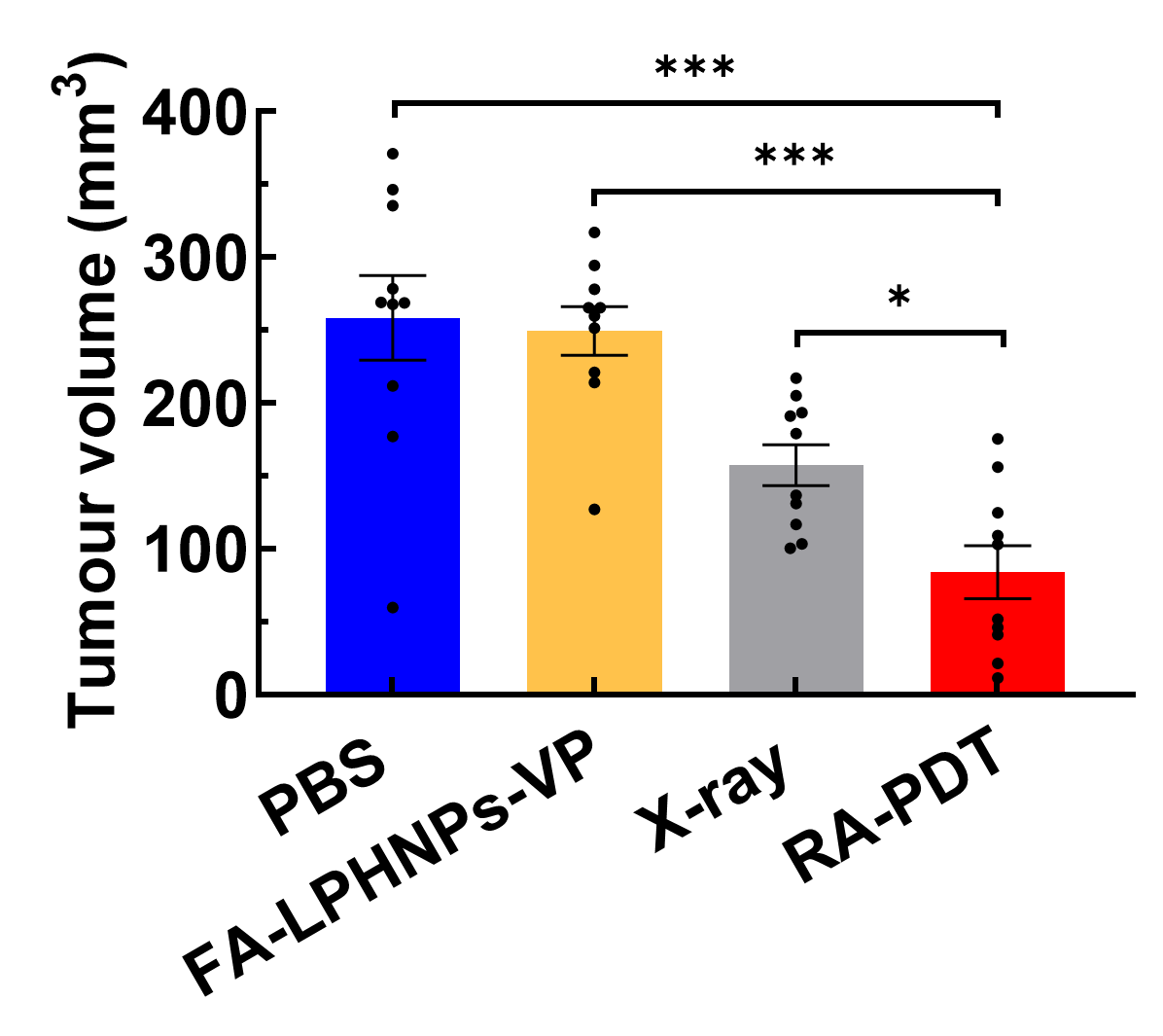


**Fig. S20** The average volumes of the primary tumours in different treatment groups at day 27 post-treatment. FA-LPHNPs-VP dose was 50 mg/kg and X-ray irradiation dose was 4 Gy (Two-way ANOVA with Tukey’s multiple comparison post hoc test, n=10 mice per group, Mean ± SEM).


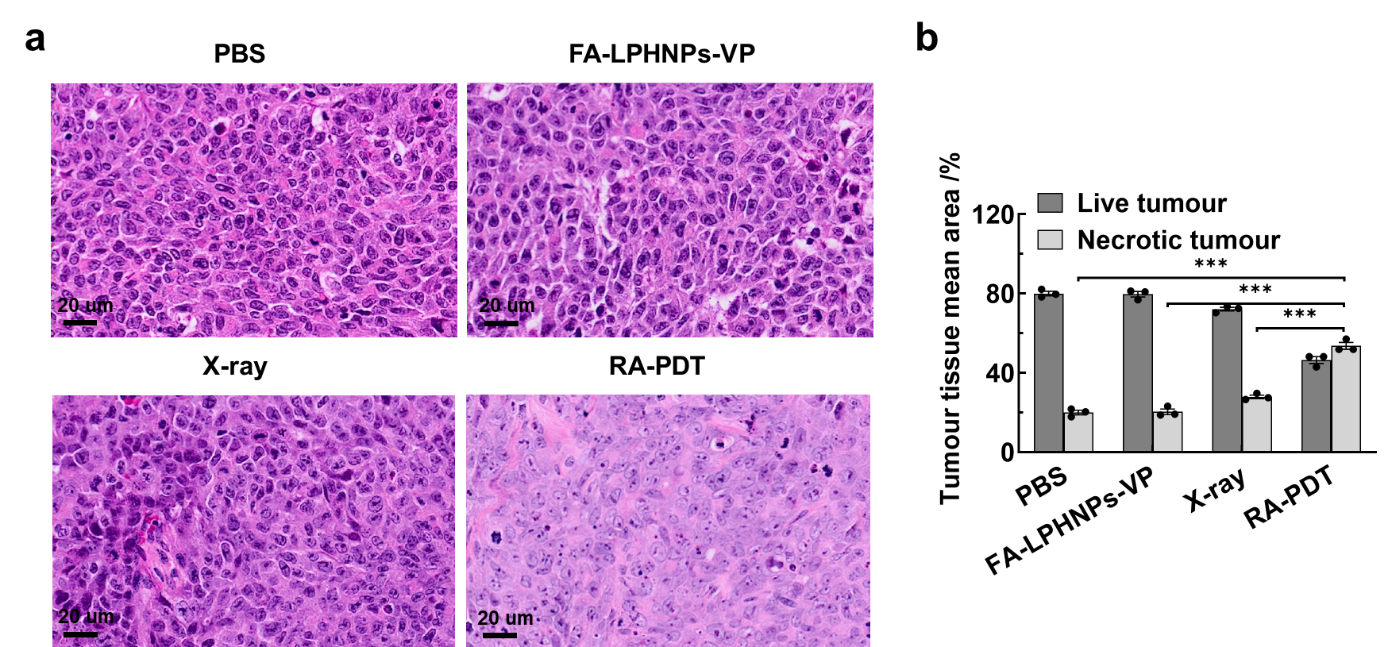


**Fig. S21** (a) Representative H&E-stained images of primary tumour tissue at day 18 post-treatment. Scale bar is 20 μm; (b) The percentage of live and necrotic cancer cells in tumour tissues in the studied groups (Two-way ANOVA with Tukey’s multiple comparison post hoc test, n=5 mice per group, Mean ± SEM), *p ＜0.05; **p ＜0.01; ***p ＜0.005.


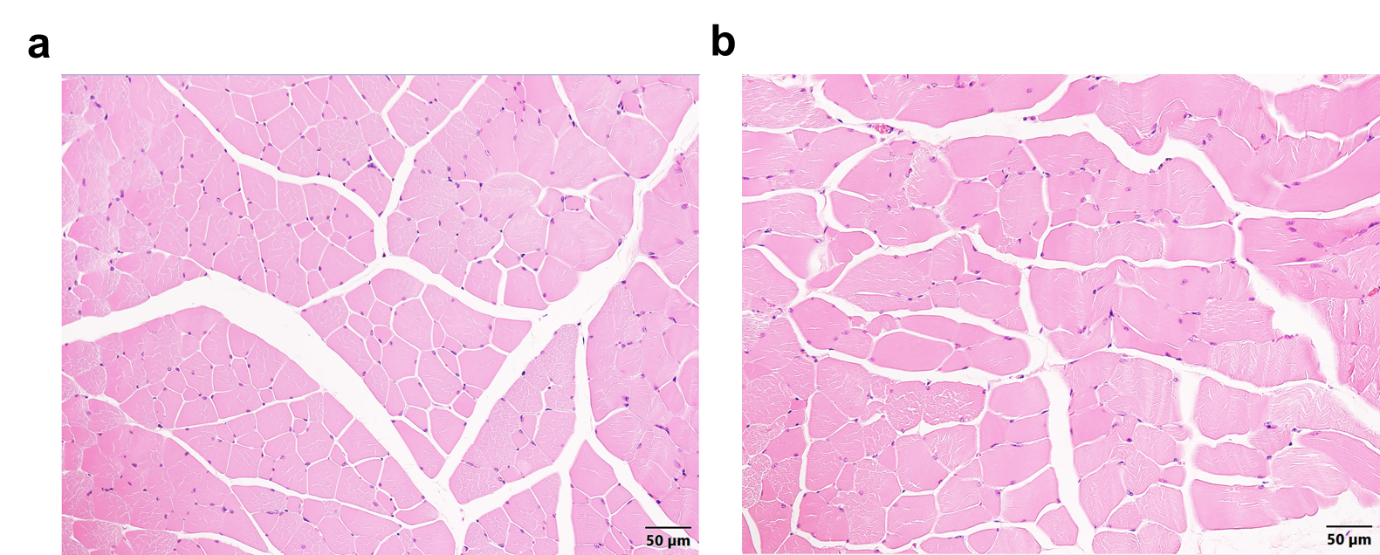


**Fig. S22** Representative H&E-stained images of the surrounding tissues of the tumour from leg muscle at endpoint from (a) PBS group and (b) X-ray irradiation group. Scale bar is 50 μm.

**Table S2** **Analysis of second tumour foci in the lymph node tissues after treatments**

| **Treatment** | **PBS** | **FA-LPHNP-VP** | **X-ray** | **RA-PDT** |
| --- | --- | --- | --- | --- |
| **Total mouse (n)** | 10 | 10 | 10 | 10 |
| **Mice with second tumour foci (n)** | 4 | 4 | 4 | 2 |
| **Second tumour foci percentage (%)** | 40% | 40% | 40% | 20% |
