# Supplementary material for: Unlocking the in vivo therapeutic potential of radiation-activated photodynamic therapy for locally advanced rectal cancer with lymph node involvement
Source: eBioMedicine. 2025 May 12;116:105724. doi: 10.1016/j.ebiom.2025.105724 (PMC12141937; doi:10.1016/j.ebiom.2025.105724)
Supplement: Animal Experimentation Approval Information [file mmc2.docx]

We clarified that all studies involving animal experimentation are ethically approved. The detailed approval information is listed as follows:

**1. Animal ethical approval:**

All animal experiments were approved by the UNSW Animal Care and Ethics Committee (project approval reference number are 20/95B, 21/39B, 21/77B, 22/30B and 23/48B).

**2. The approval body:** the UNSW Animal Care and Ethics Committee

**3. Adherence to regulatory and reporting guidelines (e.g. ARRIVE):**

NOD/SCID (6-8-week-old) and BALB/c nude mice (6-8-week-old) mice were provided by Animal Services from the Animal Resources Centre (ARC, Perth, WA). Mice were housed in specific pathogen free conditions at 22℃ with a light/dark cycle of 12 h. Mice were kept in standard ventilated cages and acclimated for one-week following arrival into the UNSW animal facility. Mice were provided food and water ad libitum and their wellbeing monitored regularly.

**4. Details about husbandry and maintenance, as well as anaesthesia and euthanasia methodology:**

**Husbandry and maintenance:**

After mice arrived, mice were kept in standard ventilated cages (no more than 5 mice/cage) and housed in specific pathogen free conditions at 22℃ with a light/dark cycle of 12 h. Mice were provided food and water ad libitum and their wellbeing monitored regularly. In addition, animals will be checked and weighed at least twice a week. Following any cell or liposome injection procedure, mice will be monitored daily for 3 days for prompt detection of any adverse events. General monitoring of behaviour, demeanour, appetite, weight, response to carers, and state of coat will be performed on the animals. The main indicator of health will be changes in the weight of the animal and should there be a weight loss of equal to or greater than 20% (of max weight), animal will be euthanised. Clinical signs indicating blockage of the colon such as changes in respiratory effort, volume and consistency of stools, and a distended abdomen will also be closely monitored for. Reductions in mobility and weight-bearing caused by tumour growth on the foot will be closely monitored for, as will any signs of pain including isolation, vocalisations and self-mutilation; to reduce occurrence of irritation at the injection site, soft bedding such as the shredded tissue nesting material available in the facility will be added to the cages. All criteria are included in the monitoring sheet; once signs of deterioration appear daily monitoring will commence, and animals will be euthanised based on the scores laid out in this sheet when a cumulative score of 5 in any single category or across all categories is obtained.

If signs of dehydration are detected, such as sunken eyes or changes in skin turgor, 200 µL of 0.9% saline will be injected subcutaneously into the mice. Following injections into the footpad, and when changes in grooming, mobility/gait, activity levels and weight scoring between 2-4 are identified, moistened food will be placed on the floor of the cage so the mice will not need to climb to the tray to reach food, encouraging consumption of feed. In addition to a weight loss of 20%, tumours greater than 100 mm^3 as well as signs of pain and an inability to weight bear on the injected foot will all result in a score of 5 and thus euthanasia of affected animals. (Foley et al. 2019. Clinical management of pain in rodents. Comparative Medicine, 69(6):468-489.)

**Anaesthesia of animals**

Induction: Anaesthesia is performed using an isoflurane gas anaesthetic machine. Throughout the procedure, oxygen is maintained at 1 litre per minute. The vaporiser is set on 2-4% isoflurane to induce anaesthesia. The safety margin of this anaesthetic agent is high and a satisfactory recovery from minor procedures will occur within a few minutes following isoflurane withdrawal. There are no anticipated side effects with this anaesthetic. Specially, animals will be placed in the induction box. It will take approximately 3-5 mins for the animal to go under. Animals will be checked for depth of anaesthesia with the toe pinch reflex.

Maintenance: Once confirmed, the animals will be transferred to the imaging stage with a nose cone to maintain the animal under anaesthesia. During procedures, respiration will be monitored to ensure an adequate depth of anaesthesia is maintained. For maintenance of anaesthesia the vaporiser is retained at between 1 and 3%. It will be adjusted depending on the breathing pattern of the mouse to avoid quick anaesthesia and overdose causing death. Active scavenging will be used for any procedures that require more than 20 mins. Once animals are anaesthetised, eye lubrication will be applied. Humidified anaesthetic gas will be used in any procedures requiring anaesthesia for longer than 20 mins.

Additional support: All instruments have a heated animal bed to maintain the animals' body temperature while they are being imaged.

Location of induction and recovery areas: Allocated anaesthetic induction boxes are located next to procedural and imaging areas. Mice are individually recovered from anaesthesia in a fresh cage until fully mobile and are then returned to their cage mates.

**Euthanasia methodology:**

Method, location and expertise of personnel: Animals will be euthanised with CO_2_ inhalation followed by cervical dislocation when the endpoint is reached. Or cervical dislocation will be carried out while animals are anaesthetised with isoflurane right after an imaging procedure. The CO_2_ euthanasia procedure will be performed as outlined in the UNSW ACEC CO_2_ Euthanasia Guideline 2020_Final document (https://research.unsw.edu.au/document/UNSW%20ACEC%20CO2%20Euthanasia%20Guideline%202020_Final.pdf) which involves a gradual increase of inhaled CO_2_. Cervical dislocation will be performed as described by Flinders University (https://staff.flinders.edu.au/content/dam/staff/research/ebi/animal/sops/swms-methods-of-humane-euthanasia-in-mice.pdf) and involves dislocating the atlanto-occipital joint by squeezing the cervical vertebrae immediately behind the skull until the vertebrae are completely separated, ensuring destruction of the brain stem. Euthanasia will take place in one of the Lowy Cancer Research Centre's Animal Facility procedure rooms away from any other animals. Research participants involved in euthanising the animals have all attended the UNSW's animal care and ethics course and have experience with euthanasia of mice.
