# Supplementary material for: Unlocking the in vivo therapeutic potential of radiation-activated photodynamic therapy for locally advanced rectal cancer with lymph node involvement
Source: eBioMedicine. 2025 May 12;116:105724. doi: 10.1016/j.ebiom.2025.105724 (PMC12141937; doi:10.1016/j.ebiom.2025.105724)
Supplement: Reagent Validation File [file mmc3.docx]

All reagents were purchased from their respective suppliers and used according to the manufactures’ protocol.

**Key resources table**

| **REAGENT or RESOURCE** | **SOURCE** | **IDENTIFIER** |
| --- | --- | --- |
| **Cell line** | | |
| HCT116 | ATCC | Cat#CCL-247  RRID:CVCL_0291 |
| CCD841 CoN | ATCC | Cat#CRL-1790  RRID:CVCL_2871 |
| HCT116-Luc2 | ATCC | Cat#CCL247LUC2  RRID:CVCL_VU38 |
| **Antibody** | | |
| Ki-67 Recombinant Rabbit Monoclonal Antibody (SP6) | Thermo Fisher Scientific Aust Pty Ltd | Cat#MA5-14520  RRID:AB_10979488  IHC |
| Rabbit monoclonal [EPR1579Y] to Cytokeratin 19 | Abcam | Cat# ab76539  RRID:AB_1523469  IHC |
| BOND Polymer Refine Detection | Leica Biosystems Pty Ltd | Cat# DS9800  RRID:AB_2891238  IHC |
| **Organisms/strains** | | |
| Mouse NOD/SCID | Animal Resources Centre (ARC, Perth, WA) | N/A |
| Mouse Balb/c nude mice | Animal Resources Centre (ARC, Perth, WA) | N/A |
| **Reagent** | | |
| LEGEND MAX™ Mouse IFN-γ ELISA Kits | BioLegend | Cat#430807 |
| LEGEND MAX™ Mouse TNF-α ELISA Kits | BioLegend | Cat#430907 |
| LEGEND MAX™ Mouse IL-6 ELISA Kits | BioLegend | Cat#431307 |
| Luminex Muse® Cell Cycle Kit | Abacus dx | Cat#LUMCH100106 |
| CellTiter 96® AQueous One Solution Cell Proliferation Assay kit | Promega | Cat#G3580 |
| Apoptosis/Necrosis Assay Kit (blue, red, green; ab176750) | Abcam | Cat#ab176750 |
| Pierce™ D-Luciferin Monopotassium Salt | Thermo Fisher Scientific Aust Pty Ltd | Cat#88294 |
| Lecithin | Avanti Polar Lipids | Cat#P7443 |
| 1,2-distearoyl-sn-glycero-3-phosphoethanolamine-N-carboxy (polyethylene glycol) 2000 (DSPE-PEG2000) | Avanti Polar Lipids | Cat#880135P |
| DSPE-PEG-Folate  (DSPE-PEG-FA) | Nanocs Inc. | Cat#PG2-DSFA-2k |
| Poly (D, L-lactic-co-glycolic acid) (PLGA) | Sigma-Aldrich | Cat#719900 |
| Verteporfin | Sigma-Aldrich | Cat#SML0534 |
| 2’7’-dichlorofluorescin diacetate (DCF-DA) | Sigma-Aldrich | Cat#D6883 |
| Acetonitrile | Sigma-Aldrich | Cat#271004 |
| ReadyProbes^TM^ Cell Viability Imaging Kit (Blue/Green) | Thermo Fisher Scientific Aust Pty Ltd | Cat#R37609 |
| Singlet Oxygen Sensor Green (SOSG) | Thermo Fisher Scientific Aust Pty Ltd | Cat#S36002 |
| NucBlue™ Live Ready Probes™ Reagent(Hoechst 33342) | Thermo Fisher Scientific Aust Pty Ltd | Cat#R37605 |
| Antibiotic-antimycotic (100X) | Thermo Fisher Scientific Aust Pty Ltd | Cat#15240062 |
| Dulbecco’s Phosphate-Buffered Saline solution (DPBS) | Thermo Fisher Scientific Aust Pty Ltd | Cat#14190250 |
| Hank’s Balanced Salt Solution (HBSS) | Thermo Fisher Scientific Aust Pty Ltd | Cat#14175145 |
| Opti-Minimal Essential Medium (Opti-MEM) | Thermo Fisher Scientific Aust Pty Ltd | Cat#31985062 |
| Invitrogen™ Live Cell Imaging Solution (1X) | Thermo Fisher Scientific Aust Pty Ltd | Cat#A14291DJ |
| McCoy’s 5A Medium | ATCC | Cat#ATC302007 |
| Eagle's Minimum Essential Medium (EMEM) | ATCC | Cat#ATC302003 |
| Foetal Bovine Serum (FBS) | Bovogen Biologicals Pty Ltd | Cat#SFBS |

IHC: Immunohistochemistry
